# Marine prebiotics mediate decolonization of *Pseudomonas aeruginosa* from gut by inhibiting secreted virulence factor interactions with mucins and enriching *Bacteroides* population

**DOI:** 10.1186/s12929-023-00902-w

**Published:** 2023-02-02

**Authors:** Rajendra Prasad Janapatla, Anna Dudek, Chyi-Liang Chen, Chih-Hsien Chuang, Kun-Yi Chien, Ye Feng, Yuan-Ming Yeh, Yi-Hsin Wang, Hsin-Ju Chang, Yuan-Chuan Lee, Cheng-Hsun Chiu

**Affiliations:** 1 Molecular Infectious Disease Research Center, Chang Gung Memorial Hospital, Taoyuan, Taiwan; 2 Department of Pediatrics, St. Paul’s Hospital, Taoyuan, Taiwan; 3 Graduate Institute of Biomedical Sciences, Chang Gung University College of Medicine, Taoyuan, Taiwan; 4 Institute for Translational Medicine, Zhejiang University School of Medicine, Hangzhou, China; 5 Chang Gung Microbiota Therapy Center, Chang Gung Memorial Hospital, Taoyuan, Taiwan; 6 Department of Biology, Johns Hopkins University, Baltimore, MD USA; 7 Division of Pediatric Infectious Diseases, Department of Pediatrics, Chang Gung Memorial Hospital, Chang Gung University College of Medicine, Taoyuan, Taiwan

**Keywords:** *Pseudomonas aeruginosa*, Two-partner secretion system, Hemagglutinin, Gastrointestinal colonization, Microbiota, Dysbiosis, Prebiotics, Fucoidans

## Abstract

**Background:**

*Pseudomonas aeruginosa* intestinal carriage rates are significantly higher in immunosuppressed individuals and hospitalized patients who therefore have increased risk of infections and antibiotic-associated diarrhea. To combat intestinal dysbiosis and decolonize *P. aeruginosa* from gastrointestinal tract, we investigated the anti-adherence and gut microbiota modulation properties of marine prebiotic fucoidans.

**Methods:**

Proteomic analysis of culture supernatant was performed by LC–MS/MS. Using lectin-based enzyme-linked immunosorbent assay, hemagglutinin domain interaction and inhibition with biomolecules were studied. We investigated the role of nutritional grade fucoidans in a mouse model and used 16S ribosomal RNA sequencing to examine fecal microbiota composition.

**Results:**

Analysis of culture supernatant proteins indicated the secretion of two-partner secretion (TPS) family proteins, including TpsA1/CdiA2 and TpsA2/CdiA1. Lectin like activity at the N-terminal of TpsA due to a conserved hemagglutinin domain (Pfam identifier [ID] PF05860) mediates binding to mucins that carry multiple fucosylated glycans. Fucose-rich sulfated polysaccharides (fucoidans) and sulfated dextrans were found to be potent inhibitors of the recombinant N-terminal hemagglutinin domain of TpsA (TpsA-NT-HAD) binding to mucins. In a mouse model, antibiotic-induced dysbiosis was essential for *P. aeruginosa* gastrointestinal colonization. After prophylactic oral fucoidans supplementation, a higher proportion (60%) of the mice were decolonized over time and resisted re-colonization, this was associated with remarkable expansion of *Bacteroides* (post-infection day-3 abundance, 29–50%) and consequential reductions in bloom of *Enterobacteriaceae* and *Enterococcaceae* populations. In the non-supplemented group, *Parabacteroides* mediated recovery from dysbiosis but failed to decolonize *P. aeruginosa*.

**Conclusions:**

Supplementing diet with marine prebiotic fucoidans can mediate earlier recovery from dysbiosis and decolonization of *P. aeruginosa* from gut by inhibiting secreted virulence factor (TpsA/CdiA) interaction with mucins and promoting the growth of beneficial *Bacteroides* population. We suggest the prophylactic use of nutritional grade fucoidans to decolonize *P. aeruginosa* from gastrointestinal tract of at-risk individuals to prevent infection and transmission of colonizing *P. aeruginosa*.

**Supplementary Information:**

The online version contains supplementary material available at 10.1186/s12929-023-00902-w.

## Introduction

*Pseudomonas aeruginosa* can be isolated from < 10% of fecal samples collected from healthy children and adults and *P. aeruginosa* intestinal carriage rates are significantly higher in immunosuppressed individuals after antibiotic treatment [1–3]. Patients colonized with *P. aeruginosa* on admission tend to develop infection due to their colonizing strain during their hospital stay [4–6]. *P. aeruginosa* can cause antibiotic-associated diarrhea among immunosuppressed or hospitalized patients and children [7–9]. Shanghai fever caused by *P. aeruginosa* in children is one of the most severe enteric diseases with complications [10]. Although prophylactic antibiotic regimens for selective decontamination of the digestive tract (SDD) improved outcomes of critical patients in settings with low prevalence of antibiotic resistance [11], appropriate regimens have not yet been determined for *P. aeruginosa*. To combat intestinal dysbiosis and decolonize multidrug-resistant (MDR) *P. aeruginosa* from gastrointestinal (GI) tract, use of antibiotics-independent methods like, fecal microbiota transplantation (FMT), probiotics, prebiotics and synbiotics have gained increased interest in recent years [12–15]. Polysaccharides from marine seaweeds, including those sulfated (fucoidan, agaran, carrageenan, and ulvan) and non-sulfated (laminaran and alginate) exhibit prebiotic properties by modulating the composition of gut microbiota (GM) and consequently, prevent certain gastrointestinal infections [16–18]. Alginates tend to increase *Bacteroides, Bifidobacterium*, and *Lactobacillus* species while laminarans mainly stimulate *Bacteroides* sp. [19]. Sulfated polysaccharides including fucoidans influence the gut microbiota composition in several ways, and the influence may vary depending on the type of fucoidan, animal models, and treatment duration [20]. In healthy C57BL/6 mice, *Laminaria japonica* treatment significantly increased the abundance of *Ruminococcaceae*, while *Ascophyllum nodosum* treatment significantly increased the abundance of *Lactobacillus* [21]. Shibata et al. reported that by supplementin*g Cladosiphon* fucoidan to Mongolian gerbils, *H. pylori-*induced gastritis was strongly suppressed and 30% of gerbils were cured of *H. pylori* infection, which may reduce the risk of associated gastric cancer [22].

Although gut microbiota acts as a barrier against intestinal pathogens, *P. aeruginosa* overcomes the resistance to colonization mediated by gut microbiota and innate immune system, by producing an impressive array of virulence factors [23–26]. *P. aeruginosa* lectins LecA and LecB and extracellular appendages, such as flagella and pili, play a major role in the attachment of bacteria to their host and have been implicated in adhesion [27, 28]. Furthermore, *P. aeruginosa* carries large protein systems that belong to two-partner secretion (TPS) family (also known as Type Vb secretion systems, T5bSS), a TPS system mainly consists of the secreted TpsA effector protein and its TpsB partner transporter. TpsA1 (CdiA2, 573 kDa) and TpsA2 (CdiA1, 361 kDa) promote bacterial competition through contact-dependent growth inhibition systems (CDI) and adhesion and biofilm formation, therefore TPS system effectors are designated as major virulence determinants that are beneficial to Gram-negative pathogens [29, 30]. The protein domains of a TpsA/CdiA include, conserved two-partner secretion (TPS) transport domain at the N-terminus, the filamentous hemagglutinin domain 1 (FHA-1), the receptor-binding domain (RBD), the Tyr/Pro-enriched (YP) domain, the second FHA domain (FHA-2), the pre-toxin (PD) domain, and the C-terminus toxin domain (CdiA-CT) [30]. High sequence diversity was found in CdiI immunity proteins that specifically neutralize cognate CdiA-CT toxins, CdiA1 [31]. Although, the predicted functions of CdiA-CT sequences were highly diverse, most CTs tend to target nucleic acids; MafB19-like deaminase domain (Pfam ID PF14437) and EndoU RNase domain (PF14436) were predominant [31]. In *P. aeruginosa* strain PAO1, CT domain in the CdiA1 (PA0041) was found to be tRNase, while in CdiA2 (PA2464) it was predicted to be an EndoU RNase. Recent evidence suggested that CdiA-CT tRNAse activity contributes to virulence against mammalian host [30].

Majority of TpsA protein structures solved were limited to N-terminal TPS domain, or CdiA-CT domains associated with toxin activities of CDI systems [32]. Although the role of N-terminal TPS domain in secretion of the TpsA proteins is well defined, its ability to function as a carbohydrate recognition domain (CRD) that could mediate adhesion was never investigated in detail in Gram-negative bacteria. Since adhesion precedes colonization and infection, use of anti-adhesion and anti-biofilm agents that interfere with the ability of the bacteria to adhere to surfaces, cells and tissues of the host can limit the infections [28]. *P. aeruginosa* strains from Shanghai fever represent a good source to discover novel adhesins, haemagglutinins and other virulence factors that could enhance the fitness of the *P. aeruginosa* isolates to cause infection. We observed that N-terminal domain of TpsA1 and TpsA2 (TpsA-NT-HAD, CdiA-NT-HAD), which can be secreted, mediates binding to mucins rich in fucosylated glycans. Fucose-rich sulfated polysaccharides (fucoidans) and sulfated dextrans were found to be potent inhibitors of the TpsA-NT-HAD. In a mouse model of gastrointestinal colonization, antibiotic induced dysbiosis was essential for *P. aeruginosa* colonization, a higher proportion of the mice were decolonized over time due to prophylactic oral fucoidan supplementation, which was associated with remarkable expansion of *Bacteroides* and simultaneous reductions in *Enterobacteriaceae* and *Enterococcaceae* populations.

## Methods

### Bacterial strains

The *P. aeruginosa* laboratory strains PA14 and PAO1 and Shanghai fever clinical isolates were collected and used as described previously by Chuang et al. [9, 10]. *P. aeruginosa* and *Escherichia coli* were routinely cultured in Luria–Bertani (LB) medium at 37 °C with shaking at 250 rpm or on LB agar plates. Strains used in this study are listed in Additional file 2: Table S1.

### Proteomics analysis

Initially in this study, we aimed to identify novel lectins and adhesins that could enhance the virulence of Shanghai fever isolates. For proteome analysis, supernatants were collected from three *P. aeruginosa* strains (two virulent Shanghai fever isolates and reference strain PA01). Briefly, overnight cultures of *P. aeruginosa* grown in 100 mL LB Medium were centrifuged at 10,000 rpm and culture pellets were suspended in 5 mL RPMI and transferred to flasks containing 95 mL RPMI and cultured further for 24 h at 37 °C. After centrifugation to sediment planktonic cells, clear culture supernatants were filtered with 0.45 µm filters. Next, Amicon 3000 kDa ultracentrifuge tubes (Millipore, USA) were used to concentrate proteins in 50 mL supernatants to 100 uL and protein samples were submitted to liquid chromatography-tandem mass spectrometry (LC–MS/MS) analysis and processed as described previously by Feng et al. [33]. We searched the proteomics data set with six specific terms lectin, hemagglutinin, agglutinin, adhesin, carbohydrate binding and glycoprotein binding. In addition, hypothetical protein sequences were submitted to BLAST search (https://blast.ncbi.nlm.nih.gov/Blast.cgi) to verify the identity to known protein and conserved domains. Proteome analysis of culture supernatants revealed 378–462 proteins per strain, including cytoplasmic, surface associated and secreted proteins including three proteins with conserved haemagglutinin (HA) domain belonging to the two-partner secretion (TPS) family (Additional file 3: Table S2). Multiple sequence alignment of the N-terminal region (1–360 amino acids) of representative TpsA proteins was performed using CLUSTAL omega (https://www.ebi.ac.uk/Tools/msa/clustalo/). Percent identify matrix was produced by online tool available at https://www.uniprot.org/align. Phylogenetic trees were constructed using online tool phylogeny.fr (http://phylogeny.lirmm.fr).

### Protein expression and purification

Recombinant N-terminal protein fragments of TpsA/CdiA and LecB monomer were expressed in *E. coli* BL21 (DE3) (RBC Bioscience, Taipei, Taiwan) and purified according to the manufacturer’s instructions for Ni2 + affinity chromatography using Nickel-Chelating Resin (Ni Sepharose 6 Fast Flow, GE Healthcare, Sweden). Protein preparations were evaluated by sodium dodecyl sulfate polyacrylamide gel electrophoresis (SDS–PAGE) and Coomassie blue staining or by Western blotting (see detailed description in the Additional file 1: Supplementary Methods; Additional file 4: Table S3).

### Lectin based enzyme-linked immunosorbent assays (ELISA)

ELISAs were adapted and performed as previously described by Torode et al. and Wu et al. [34, 35]. Using ELISA, we examined TpsA-NT-HA domain interaction with and inhibition by different biomolecules coated on 96-well plates including, mucins, glycoproteins, neoglycoproteins, polysaccharides, dendrimers synthetic glycosides (monovalent and multivalent) and HBGAs. Fucoidans from different species (*Ascophyllum nodosum, Fucus vesiculosus, Macrocystis pyrifera, Laminaria japonica, Undaria pinnatifida* were from commercial sources (Carbosynth (UK), Sigma (USA), Marinova (Australia)) (Additional file 5: Table S4). Tris buffers were prepared as described earlier by Heimburg-Molinaro et al. [36]. Substrates in 100 μL tris saline buffer (TSB) including, porcine gastric mucin type III (PGM, 1–2 μg), fucoidans (100 μg), alginates (1–100 μg based on solubility) and histo-blood group antigens (HBGAs) (1–100 μg) were coated in a 96-well microtiter plate (Costar, USA) for 18 h at 4 °C. After blocking with TSB + 0.05% Tween-20 + 1% BSA for 1 h at room temperature (22 °C), wells were filled with serially diluted TpsA-NT-HA with or without inhibitors and incubated for 1 h. After washing, wells were incubated with a 1:5000 dilution of rabbit anti-his tag monoclonal antibody (Bioman, Taiwan) for 1 h and subsequently with 1:5000 dilution of horseradish peroxidase-conjugated goat anti-rabbit antibody (Millipore, USA) for 1 h. The color was developed using 3, 3′, 5, 5′-Tetramethylbenzidine (TMB) substrate (NeA-Blue, Clinical Science Products, USA), the change in absorbance was determined at 650 nm or at 450 nm after stopping the reaction by sulfuric acid. Based on half maximal inhibitory concentration (IC50) concentrations, high affinity and low affinity ligands were identified and analyzed further. The IC50 values were estimated by using a free online program from AAT Bioquest (https://www.aatbio.com/tools/ic50-calculator). Fucosylated glycans on porcine gastric mucin and fucoidans were identified with monoclonal antibodies and plant lectins using ELISA (see detailed description in the Additional file 1; Additional file 6: Table S5 and Additional file 7: Table S6). In vitro cell adhesion and inhibition assays were performed using human cell lines (A549 and Caco-2 cells) [10].

### Animal studies

A mouse model was used to confirm the potential prophylactic activity of fucoidans against virulent Shanghai fever strain *P. aeruginosa* S8 gut colonization [9, 10]. Five-week-old mice (five mice per group) weighing 20–25 g were used in the experiments. Mice were pretreated with clindamycin 0.6 mg intramuscularly daily for 5 days before bacterial challenge to clear the native microbiota and establish colonization. A total of 0.1 mL of 0.45% sterile saline with or without a bacterial inoculum of 1 × 10^8^ colony forming units (CFUs)/mL was delivered to the mice through an orogastric tube daily for 3 days. Nutritional grade fucoidans 0.5% (w/v), from *Fucus vesiculosus* (FV) and *Ascophyllum nodusum* (AN) (Marinova, Australia), were supplemented in the drinking water of mice for 19 days, including 2 days before, 3 days during and 14 days after orally challenging *P. aeruginosa* S8. In the positive control group (Water), mice were given drinking water before and after orally challenging with *P. aeruginosa* S8. While, mice without fucoidans supplementation and no challenge with *P. aeruginosa* S8 served as negative control (NC). To monitor bacterial shedding, stool samples were collected daily from individual mice by anal stimulation and plated on *Pseudomonas* isolation agar (Becton, Dickinson and Company, USA). Following fucoidan supplementation and oral challenge with *P. aeruginosa,* mice were considered completely decolonized of *P. aeruginosa* from GI tract when no fecal counts were observed on *Pseudomonas* isolation agar plates. A mouse was considered partially decolonized of *P. aeruginosa* when the fecal counts over time were found to be lower than those that had been ingested. The mice were observed daily for 30 days after bacterial inoculation. The animal study was approved by the Institutional Animal Care and Use Committee (CGU 108–172), Chang Gung University, Taoyuan, Taiwan.

### 16S rRNA sequencing and analysis

To investigate the role of fucoidans in bacterial dysbiosis, changes in microbiota profile due to virulent *P. aeruginosa* S8 colonization of the gut in mice were identified after analyzing the 16S rRNA sequencing data. Stool samples were collected from mice at several time points during the experiment for 16S rRNA sequencing (see detailed description in the Additional file 1: Methods) [37].

### Statistical analysis

Statistical analysis was performed by using SPSS software version 20.0 (SPSS, Chicago, IL, USA) and GraphPad Prism 5.0 (GraphPad Software, Inc., San Diego, CA, USA). Significant differences in adhesion due to incubation with fucoidan was assessed using Chi Square test. A Kaplan–Meier survival analysis (log-rank test) was used to compare decolonization over time between the fucoidan supplemented groups and control group. Significant reductions in fecal *P. aeruginosa* load (median values of CFUs) among the groups during the study period was determined using Wilcoxon signed-rank test. The paired t-test analysis was performed to compare microbiota composition between groups at different levels (phyla and genus). A *P* value of < 0.05 was considered statistically significant [38–40].

## Results

### Two-partner secretion (TPS) family proteins

By proteome analysis, a conserved hemagglutinin domain (HA, Pfamidentifier [ID] PF05860) was found at the N-terminal between 31 and 168 amino acid residues in one protein assigned as putative hemagglutinin (PA0041) and two proteins annotated as hypothetical proteins (PA2462 and PA4625) in all *P. aeruginosa* strains (Additional file 3: Table S2). Further analysis revealed that the three proteins with conserved HA domain belong to the TPS family, which are widespread in Gram-negative bacteria and consist of a large secreted exoprotein (TpsA) and a transporter protein (TpsB) located in the bacterial outer membrane. *P. aeruginosa* strains that belong to “PAO1” and “PA14” groups harbor at least five ubiquitous TPS systems, designated in PAO1 as Tps1: PA2462–PA2463 (CdiA2); Tps2: PA0040–PA0041 (CdiA1); Tps3: PA4624–PA4625 (CdrA); Tps4: PA4540–PA4541 (LepA) and Tps5: PA0690–PA0692 (PdtA) [25]. Additionally, we observed a remarkable sequence similarity (> 99%) between TpsA1 and TpsA2 in the N-terminal region (1–360 amino acids); which includes conserved hemagglutinin domain and TPS domain (Table 1; Additional file 8: Fig. S1).

**Table 1:** Protein sequence identity of N-terminal residues (1–360aa) of TpsA proteins in *Pseudomonas aeruginosa*

% Identity	Tps1: PA2462	Tps2: PA0041	Tps3: PA4625	Tps4: PA4541	Tps5: PA0690	Tps6: PA4082
Tps1: PA2462 CdiA2	100.00	99.17	24.11	23.20	23.31	19.61
Tps2: PA0041 CdiA1	99.17	100.00	23.72	23.60	23.31	20.00
Tps3: PA4625 CdrA	24.11	23.72	100.00	36.87	27.02	32.01
Tps4: PA4541 LepA	23.20	23.60	36.87	100.00	29.73	56.23
Tps5: PA0690 PdtA	23.31	23.31	27.02	29.73	100.00	27.48
Tps6: PA4082CupB5	19.61	20.00	32.01	56.23	27.48	100.00

Percent identify matrix was produced by online tool available at https://www.uniprot.org/align. Amino acid sequence alignment of the TpsA1 and TpsA2 NT domains (1–360 aa) harbored in strains PAO1 (PA2462, PA0041) and PA14 (PA14_32790, PA14_00510) revealed that the sequence identity was > 99%

### Secretion and expression of N-terminal domain (1–360aa) of TpsA1 protein

**Fig. 1:**
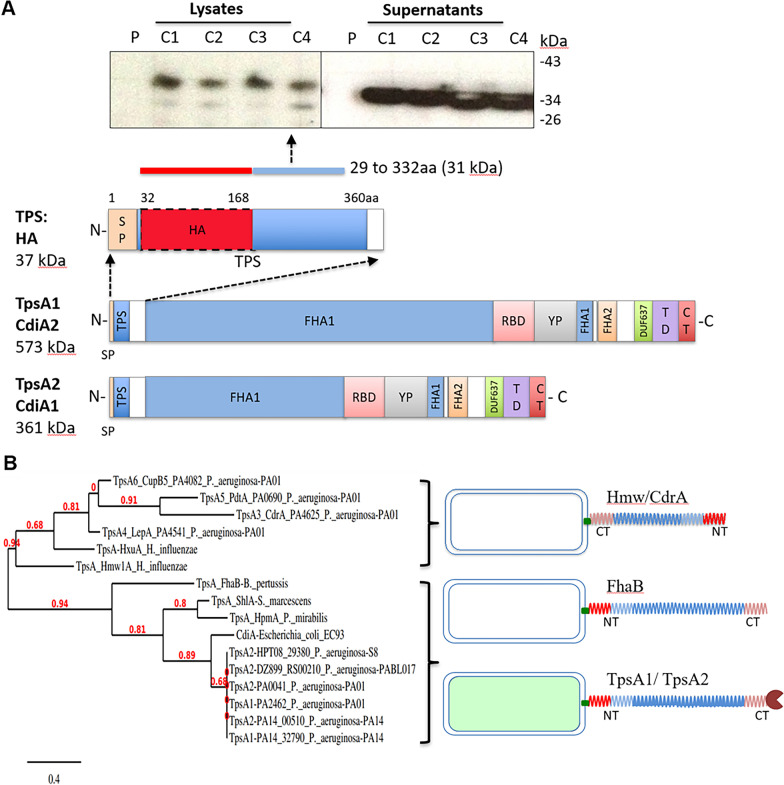
TpsA/CdiA protein domains in *P. aeruginosa.* **A** The domains of a CdiA from the N-terminus are the conserved two-partner secretion (TPS) transport domain, filamentous hemagglutinin domain 1 (FHA-1), receptor-binding domain (RBD), Tyr/Pro-enriched (YP) domain, second FHA domain (FHA-2), the pre-toxin (TD) domain, and the C-terminus toxin domain (CdiA-CT). Figure was modified from a diagram by Allen et al. [31]. A conserved region found at TpsA N-terminal termed “TPS domain” is necessary for interaction to and secretion by TpsB. Secretion of *P. aeruginosa* N-terminal domain (1–360 aa) of proteins TpsA1 and TpsA2 (TpsA-NT) expressed in *E. coli* was confirmed by immunoblot analysis of culture supernatants collected from different clones with anti-Histidine tag antibody. C1, C2, C3, C4 are clones expressing and secreting recombinant protein TpsA-NT (31 kDa) in *E. coli* BL21 (DE3). P, represents plasmid pET29b + backbone in *E. coli* BL21 (DE3). **B** Phylogenetic tree of TPS domains of TpsA NT proteins. The tree shows the subdivision of TpsA proteins into two different families Fha30-like proteins and HMW1-like proteins. Phylogenetic trees were constructed using online tool phylogeny.fr (http://phylogeny.lirmm.fr/). N terminal amino acid residues of TpsA proteins (TpsA1 to 6) of *P. aeruginosa* were compared with *H. influenzae* HMW1 and HxuA proteins; *B. pertussis* FHA protein; *S. marcescens* ShlA protein; *P. mirabilis* HpmA protein and *E. coli* CdiA protein*.* N-terminal domain is anchored to the bacterial surface and C-terminal proteins are exposed in TpsA1/CdiA1 and TpsA2/CdiA2 proteins

Although extremely large proteins TpsA1 and TpsA2 are recognized as surface associated, our analysis of *P. aeruginosa* culture supernatant proteins showed that both TpsA1 and TpsA2 are efficiently secreted into the culture medium (Additional file 2: Table S2). Therefore, we investigated whether 1–334 amino acid residues of *P. aeruginosa* TpsA (TpsA-NT31, calculated mol.wt. is 31 kDa without signal peptide) were sufficient for secretion. Since TpsB is essential for TpsA secretion, both *P. aeruginosa* proteins TpsB (full length) and TpsA-NT (31 kDa) were co-expressed in *E. coli* by inducing with IPTG. Western blot analysis with anti-histidine-tag antibodies to detect secreted and intracellular TpsA-NT31 indicated that TpsA-NT31 was secreted abundantly into the surrounding medium within 4 h after induction and only a minor quantity of TpsA-NT31 remained intracellular (with signal peptide, 34.8 kDa) or bound (mature, 32 kDa) to the cells (Fig. 1A). N-terminal signal peptide present in secreted proteins is usually sufficient to target proteins to the inner membrane and translocation across the inner membrane [41]. Both proteins TpsB and TpsA co-expressed in this study from plasmid in *E. coli* BL21 contain signal peptide sequences necessary to target the inner membrane and export. During translocation, the signal peptidase cleaves off the signal peptide, consequently TpsA-NT protein detected in the culture supernatants could be without the signal peptide.

**Fig. 2:**
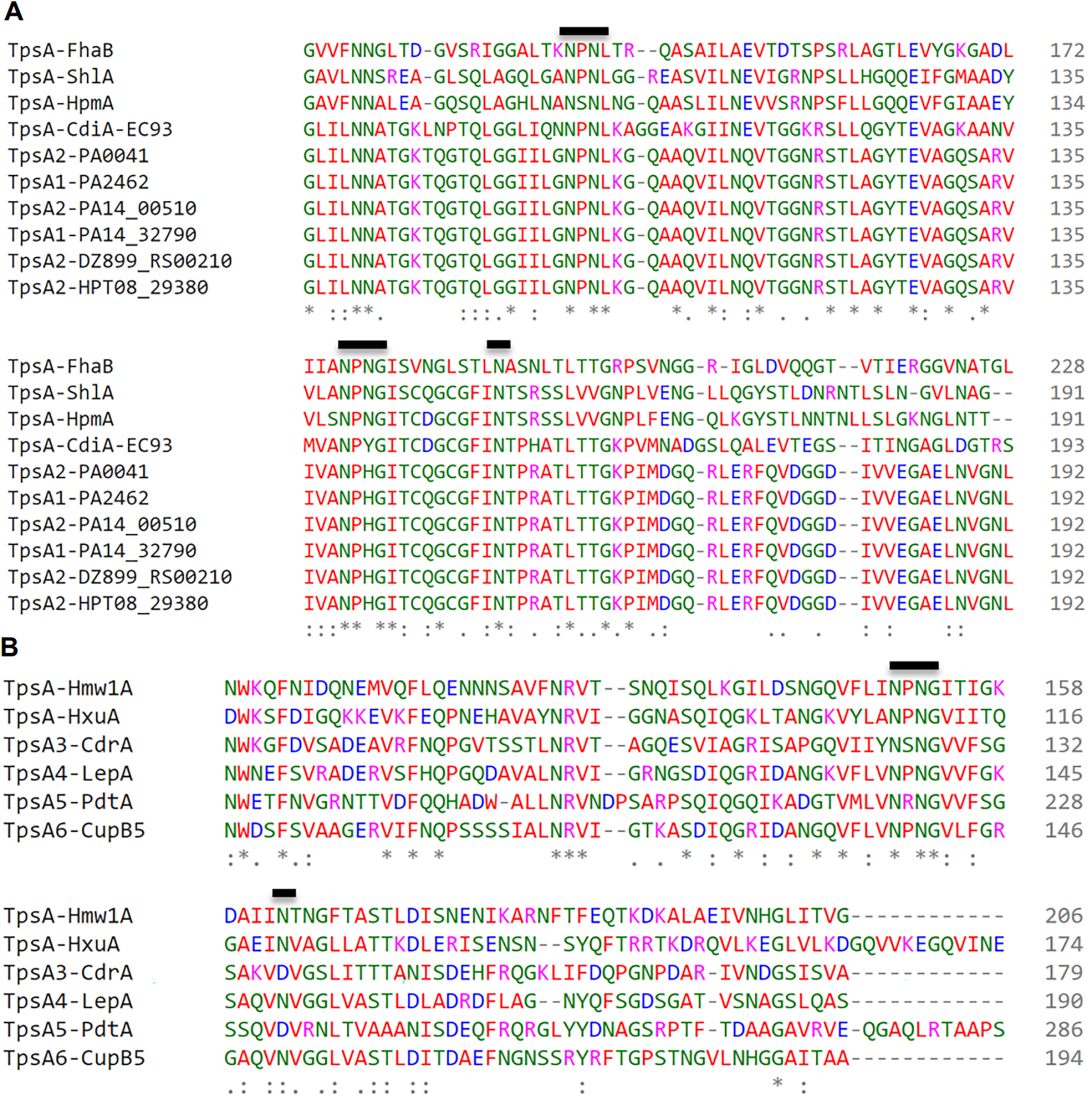
Multiple sequence alignment of the N-terminal region (1–360 aa) of representative TpsA proteins using CLUSTAL omega (https://www.ebi.ac.uk/Tools/msa/clustalo/). The numbering on the left corresponds to the amino acid number for each protein. N terminal amino acid residues of TpsA proteins (TpsA1 to 6) of *P. aeruginosa* were compared with, *B. pertussis* FHA protein (**A**) and *H. influenzae* HMW1 protein (**B**). Bold line indicates the position of motifs that have been identified in N-terminal TPS domains that are important for the secretion of TpsA proteins

All TpsA proteins share a conserved 250-residue-long N-terminal TPS domain, which is the minimal region essential for secretion of TpsA proteins. TpsB partners belong to outer-membrane proteins of the Omp85 family and the polypeptide transport-associated (POTRA) domains of several TpsB proteins have been shown to specifically recognize the TPS domains of their respective TpsA partners [32, 42]. For the amino acid sequence alignment of TPS domains compared, FHA/FhaC of *Bordetella pertussis*, ShlA/ShlB of *Serratia marcescens*, HMW1/HMW1B and HxuA/HxuB of *Haemophilus influenza,* and CdiA/CdiB of *E. coli,* which are among the few functionally well-characterized TpsA/TpsB pairs in the TPS family. Fha30-like proteins (TpsA1 & TpsA2) and HMW1-like proteins (TpsA3/4/5/6) are the two subfamilies of TPS domains that have been classified on the basis of protein sequence alignments and appear as distinct groups in the phylogenetic tree of TPS domains (Fig. 1B and Fig. 2). Three motifs have been identified in N-terminal TPS domains that are important for the secretion of TpsA proteins including NPNL box, NPNG box and a single conserved asparagine position [43]. Although all TpsA proteins contain the NPNG motif, only FHA like proteins contain the NPNL motif, but not HMW1. Both *P. aeruginosa* and *E. coli* TpsA proteins that are involved in CDI system contain all three conserved motifs necessary for secretion.

### TpsA-NT-HA domain interaction with mucins and inhibition by sulfated glycans

Since the functional significance of T5SS-NT-domain in *P. aeruginosa* and the role of HA domain in TpsA in general and CdiA in particular remain unknown, we hypothesized that *P. aeruginosa* TpsA-NT-HAD may mediate or initiate adhesion to host cell glycans and mucins. Since TpsA-NT-HA domain in TpsA1 and TpsA2 proteins are 100% identical, only TpsA1-NT- (49–332aa, 29 kDa) was expressed in *E. coli* and purified.

After preliminary screening of recombinant TpsA-NT-HA domain with diverse glycoproteins and polysaccharides using lectin-based ELISA assay, TpsA-NT-HAD was found to interact only with PGM and human ovarian cyst mucins that contain human blood group related glycans (ABH/Lewis type) (Additional file 9: Fig. S2). Characterization of PGM with fucosylated blood group glycans specific antibodies (anti-HBGA MAbs) revealed the presence of multiple HBGAs including blood group A and Lewis-Y, which were predominant glycans present in PGM (Additional file 11: Fig. S3, PGM). On the basis of the lectin reactivity to the PGM in ELISA assays, we observed that glycans specific to lectins AAL (*Aleuria aurantia*), UEA (*Ulex europaeus*), DBA (*Dolichos biflorus*), ConA (*Canavalia ensiformis*) and LTL (*Lotus tetragonolobus*) are predominantly present on PGM (Additional file 12: Fig. S4, PGM). However, only LTL that specifically binds to α1,3-fucosylated glycans such as Lewis-X, Lewis-Y and 6′sulfo sialyl Lewis-X could inhibit the interaction of T5SS-NT to PGM in a dose dependent manner (IC50, 17.564 μg/mL, Table 2) [44].

**Table 2:** Comparison of average IC50 values and potency of polysaccharides to inhibit TpsA-NT-HAD binding to porcine gastric mucin type III obtained by ELISA

Inhibitors	IC50 average µg/mL	Range (µg/mL)	SD	Potency vs	
				Porcine Gastric Mucin = 1	Chondroitin sulfate = 1
Dextrans					
Dextran sulfate-500 kDa	0.018	0.001–0.066	0.021	1892.000	25,909.536
Dextran sulfate 6–11 kDa	0.194	0.046–0.962	0.242	171.926	2354.401
Dextran 450 kDa	NSI	NSI	NSI	NSI	NSI
Dextran 9–10 kDa	NSI	NSI	NSI	NSI	NSI
Fucoidans and mucin					
*Macrosystis pyrifera*	0.277	0.113–0.423	0.096	120.529	1650.556
*Undaria pinnatifada*	0.430	0.183–9.77	0.252	77.600	1062.674
*Fucus vesiculosus* crude	0.491	0.111–1.54	0.318	67.947	930.479
*Ascophyllum nodusum* nutri. 0.5%	0.736	0.405–1.29	0.335	45.352	621.067
*A. nodusum* + *L. digitata* (PS-II)	0.764	0.113–0.423	0.096	43.649	597.734
*Fucus vesiculosus* 95%	1.008	0.21–2.797	0.760	33.107	453.375
κ-carrageenan	1.101	0.667–1.883	0.390	30.301	414.948
*Fucus serratus*	1.520	0.203–3.099	0.796	21.954	300.648
*F. vesiculosus* HCl 10′ 30 kDa	1.690	0.1–2.828	0.685	19.749	270.443
*F. vesiculosus* Aus. 30 kDa	2.547	0.656–7.066	0.501	13.100	179.396
*F. vesiculosus*—nutri. 0.5%	2.924	0.331–9.67	3.908	11.411	156.262
*F. vesiculosus* HCl 10' mix	5.468	3.215–5.215	1.520	6.102	83.568
*Laminaria digitata*	5.593	0.227–21.0	5.983	5.966	81.698
*L. japonica*	21.103	9.67–44.447	9.806	1.581	21.654
Porcine Gastric Mucin Type III	33.368	10.156–75.117	15.115	1.000	13.694
*A. nodusum*	39.886	7.51–71.197	18.179	0.837	11.456
*F. vesiculosus* HCl 60′ 30 kDa	59.022	13.92–113.723	34.505	0.565	7.742
*F. vesiculosus* HCl 60' mix	68.958	48.184–86.822	23.351	0.484	6.627
l-Fucose, d-Fucose, Fuc-BSA, methyl-b-Fuc, methyl-a-Fuc	NSI	NSI	NSI	NSI	NSI
Glycosaminoglycans					
Chondroitin sulfate-Sigma	456.950	369.911–674.852	87.803	0.073	1.000
Heparin, heparan sulfate, keratan sulfate	NSI	NSI	NSI	NSI	NSI
Alginates					
*A. nodusum* alginate	3.587	1.243–7.81	2.210	9.301	127.375
Alginic acid	4.931	0.975–13.49	5.053	6.767	92.665
*L. japonica* alginate	34.228	15.439–55.329	11.284	0.975	13.350
* Azotobacter* spp. alginate	NSI	NSI	NSI	NSI	NSI
Others					
Lotus lectin (LTL)	17.564	13.943–27.717	8.408	3.968	26.015
Sambucus Nigra lectin (SNA)	52.924	42.695–631.54	14,466	2.306	8.633
Concanavalin A lectin (ConA)	140.943	79.19–237.597	84.774	0.393	3.242
Blood Group Lewis-B	471.548	7.919–23.760	60.393	0.552	0.969

NSI: no significant inhibition (IC50 > 1000 µg/mL); Fuc: fucose, nutri.: nutritional grade fucoidan*.* The IC50 values were estimated by using a free online program from AAT Bioquest (https://www.aatbio.com/tools/ic50-calculator)

**Fig. 3:**
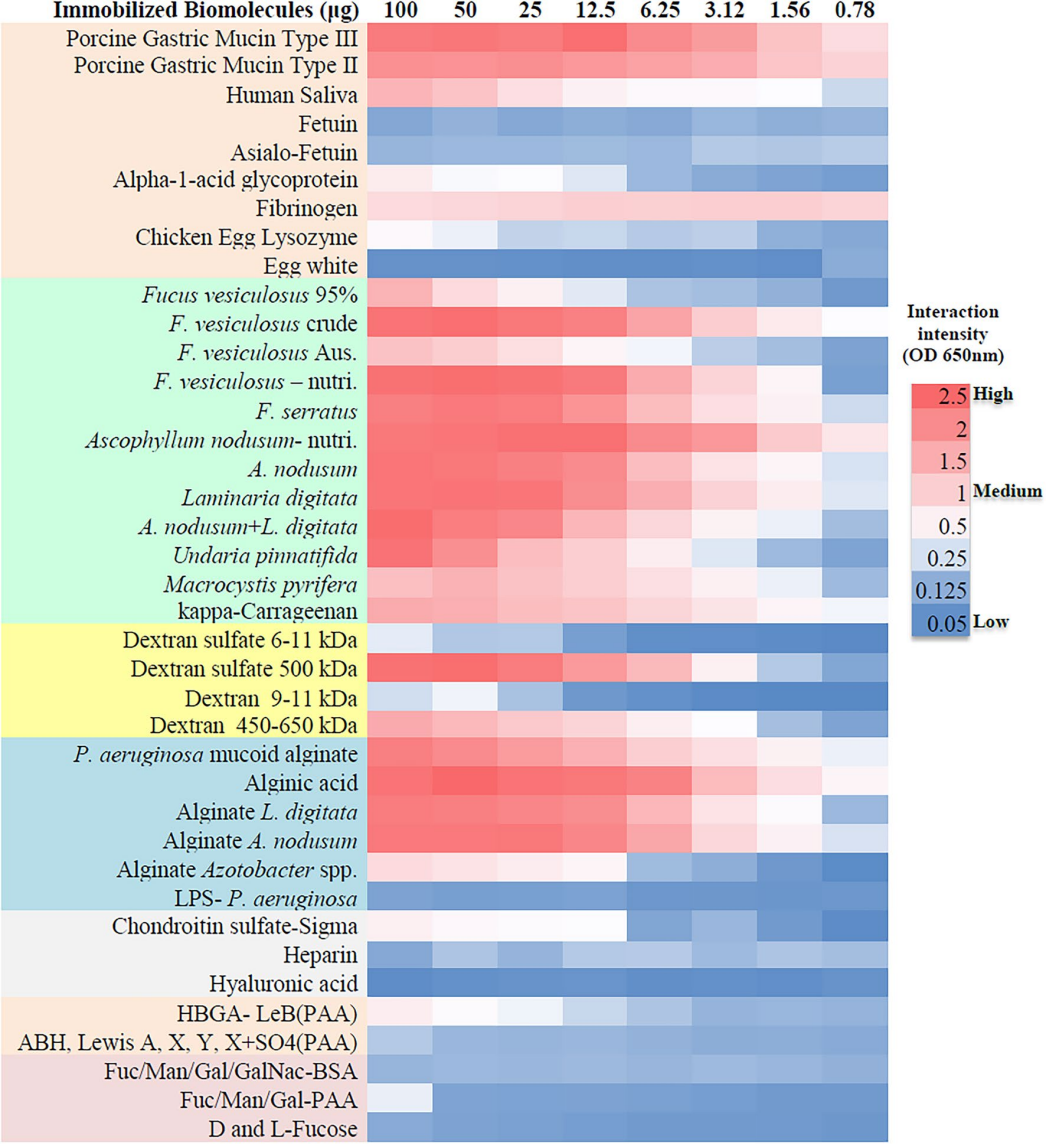
Representative results for interaction between TpsA-NT-HA domain and immobilized biomolecules. A color gradient heat map, with high reactivity (Red) to no reactivity (Blue) based on lectin ELISA OD_650_ values, has been applied to the well values. Different groups of biomolecules also colored

As shown in Fig. 3, TpsA-NT-HAD mostly bound to mucins, sulfate and fucose-rich polysaccharides (fucoidans), sulfated dextrans and marine alginates. Among the blood group glycans tested, it only bound moderately to Lewis-B. Since immobilization efficiency of the polysaccharides may not be the same, inhibition assays were also performed with the polysaccharides, which might represent the interactions between protein and polysaccharide more accurately. Preincubation of TpsA-NT-HAD with various concentrations of biomolecules inhibited the binding to PGM in a dose-dependent manner, fucoidans and sulfated dextrans were the best ligands for TpsA-NT-HAD, followed by marine alginates (Table 2), which indicates specific interaction between TpsA-NT-HAD and PGM. Depending on species, source and season structure of fucoidans vary significantly in branching, linkages (1 → 3) & (1 → 4), sulfation and acetylation, we examined the inhibitory effects of different fucoidans on T5SS-NT-HAD by binding and inhibition studies [45]. Among the fucoidans tested, *M. pyrifera, U. pinnatifada, F. vesiculosus* crude, *A. nodusum* nutritional grade and PS-II were the best inhibitors (IC50, < 1 μg/mL) of interaction between TpsA-NT-HAD and PGM. *F. vesiculosus* 95%, *F. serratus*, and kappa-Carragenan also showed good inhibitory effect (IC50, 1–1.52 μg/mL). Fucose specific lectin LecB interaction with PGM was not inhibited by fucoidans (Additional file 10: Table S7). In an intestinal cell-based model, adhesion of *P. aeruginosa* S8 to Caco 2 cells was reduced in the presence of fucoidans in a dose-dependent manner (Additional file 13: Fig. S5 and Additional file 14: Fig. S6). Characterization of fucoidans with lectins revealed that AAL specific glycans were predominant followed by MAL2 and CONA (Additional file 12: Fig. S4, FV & FS). Fucoidans could mimic sialyl Lewis-X, 6’sulfo sialyl Lewis-X, sialyl Lewis-A, Lewis-B and Lewis-Y glycans on the basis of their reactivities to the anti-HBGA MAbs (Additional file 11: Figs. S3, FV & FS).

We predicted that high molecular weight and sulfation level are two important factors for sulfated polysaccharides to be effective inhibitors. We did observe de-sulfation and depolymerization of *F. vesiculosus* due to mild acid hydrolysis resulted in reduced inhibition or no inhibition of TpsA-NT-HAD, fractions collected after fucoidan was treated with acid for 10 min showed reduced inhibition while the fractions collected after 60 min showed poor inhibitory effect, consequently sequential decrease in potency of *F. vesiculosus* was observed due to treatment and purification processes (Table 2; Additional file 15: Fig. S7 and Additional file 16: Fig. S8).

Notably, importance of sulfation on polysaccharides was further confirmed when both low and high molecular weight dextran sulfates (DS), which are also considered as analogues of the glycosaminoglycans (GAGs), could inhibit the biding activity of TpsA-NT at nanogram concentrations. Average IC50 values for DS-500 kDa and DS 6–11 kDa were 18 ng/ and 194 ng/mL, respectively. Marine alginates like *A. nodusum* alginate (9.301) and alginic acid (6.767) were more potent than PGM, while *L. japonica* alginate potency (0.975) was similar to PGM. Among the blood group glycans tested TpsA-NT could consistently interact only with Lewis-B antigen conjugated to PAA, but inhibition was observed only at high concentrations (IC50, 471.548 μg/mL).

### Prophylactic prebiotic fucoidans to reverse bacterial colonization in a mouse model

**Fig. 4:**
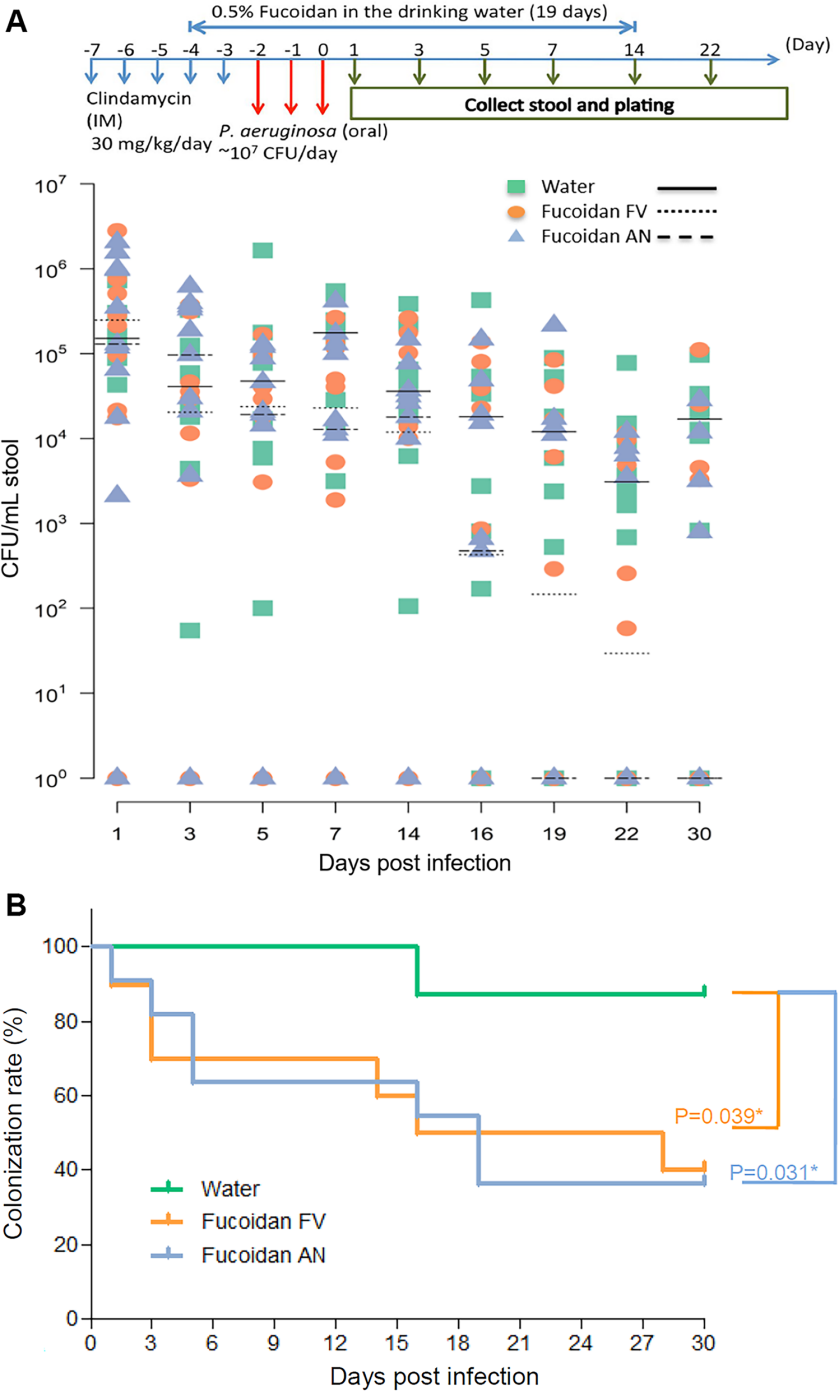
Duration of fecal shedding of *P. aeruginosa* after prophylactic fucoidans-supplementation. **A** Timelines of fecal shedding tests were scheduled for 30 days, including 2 days before and 14 days treatment of fucoidans FV (*Fucus vesiculosus*) and AN (*Ascophyllum nodusum*) after challenge of Shanghai-fever strain of *P. aeruginosa*. Negative control (NC) was the mouse challenged with water alone. Oral challenge of *P. aeruginosa* (1.0 × 10^7^ CFU) was applied for three days from Day-2 to Day 0. Fucoidans FV, and AN (0.5% in water, w/v) were used to feed mice (10 mice per each group) from Day-4 to Day + 14. The horizontal bars indicate median values of CFUs in stool of individual animals at different points in time (days post-infection). (**B**) Kaplan–Meier curves displaying *P. aeruginosa* decolonization rates after treatment of fucoidans FV (*Fucus vesiculosus*) and AN (*Ascophyllum nodusum*). *P. aeruginosa* detection in stool was considered positive carrier. *P. aeruginosa* colonization rates in the gastrointestinal tract at the end of the study in mice were significantly different between the groups with and without fucoidan treatment

Among the mice that were fed with fucoidans for 14 days p.i., two patterns of decolonization were observed: (1) *P. aeruginosa* was eliminated from a higher proportion of the mice with time, (2) but in the remaining mice *P. aeruginosa* persisted throughout the study period but with declining bacterial loads over time (Fig. 4A). Decolonization of *P. aeruginosa* from GI tract was evident within first week of p.i., in 45% of *A. nodusum* fed mice and 30% of *F. vesiculosus* fed mice (Fig. 4B; Additional file 17: Table S8). On day 14 p.i., which was the last day of fucoidan feeding, around 45% of mice were decolonized. Of note, between p.i. day 15 and day 30 among the fucoidan fed groups proportion of the mice that were decolonized further increased to 60% (P < 0.05). Whereas in the untreated mice even at p.i. day 14, decolonization was not observed, however between day 15 and day 30 decolonization was found in only < 15%.

Among the mice that persisted with *P. aeruginosa* in the GI tract, bacterial counts gradually declined significantly from p.i. day 3 through day 30 in both the fucoidan fed groups (Day 1 vs. day 3 to 30) (Fig. 4B). Remarkably, in the fucoidan fed groups mice that were once decolonized were found to be resistant to further re-colonization despite housing one group in one cage, indicating re-colonization resistance by altered gut microbiota (Additional file 18: Fig. S9).

### Reversal of gut dysbiosis by fucoidans

**Fig. 5:**
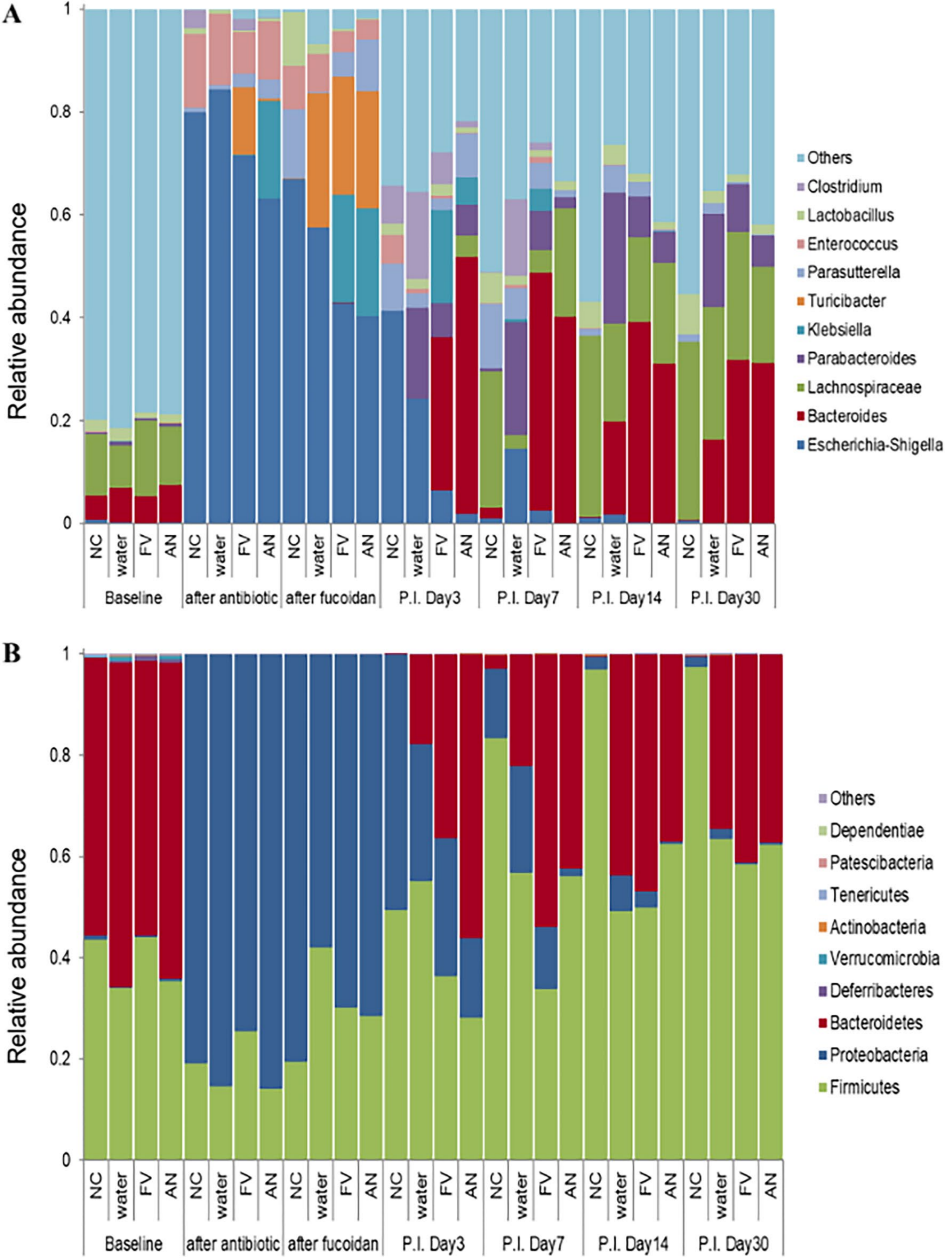
Relative abundance at the genus level (**A**) and phylum level (**B**) between fucoidan treatment groups and untreated groups. Fucoidans treatment groups FV (*Fucus vesiculosus*) and AN (*Ascophyllum nodusum*). Controls were untreated water only (W) and uninfected negative controls (NC). The paired t-test analysis was performed to compare microbiota composition between groups at different levels (phyla and genus)

**Fig. 6:**
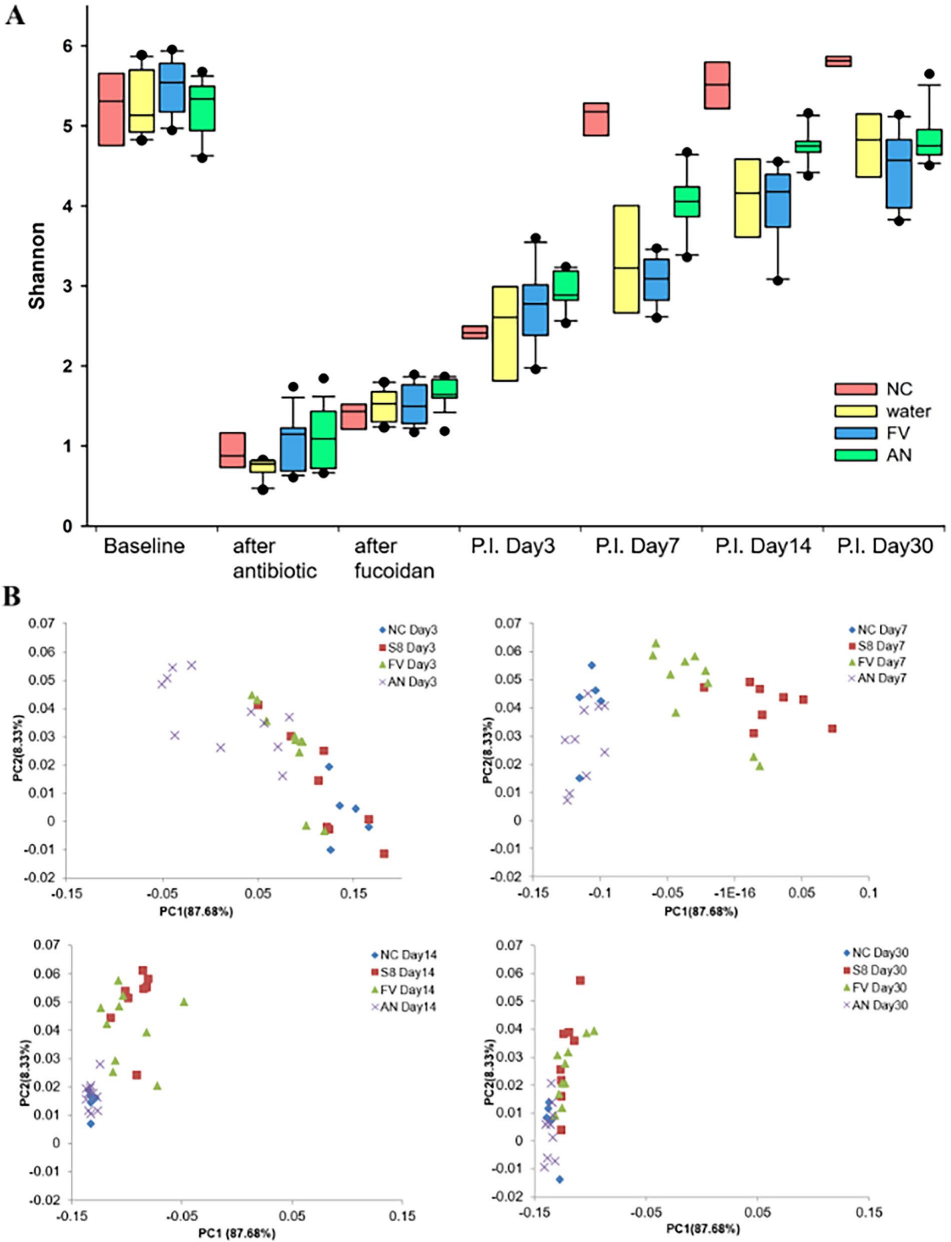
Bacterial diversity in the fecal microbiota of the study groups. **A** Shannon index of alpha diversity in the gut microbiota were compared among the study groups. **B** Principal coordinates analysis (PCoA) 2D plots for beta diversity in the fecal microbiota of the study groups. The beta-diversity of microbiota was compared using the PCoA plot based on Bray–Curtis distance distances. Fucoidans treatment groups FV (*Fucus vesiculosus*) and AN (*Ascophyllum nodusum*). Controls were untreated water only (W) and uninfected negative controls (NC)

We observed that GI tract colonization by *P. aeruginosa* in healthy mice was possible only after antibiotic treatment, therefore commensal bacteria residing in GI tract could mediate colonization resistance against *P. aeruginosa* S8 including, *Lachnospiraceae_NK4A136_*group, *Bacteroides* and *Ruminococcaceae*_UCG-014 which were predominant bacterial genera (relative abundance ≥ 10% each) found in the fecal microbiota (Fig. 5A). After a 5-day course of intramuscular administration of clindamycin (0.6 mg/day) to naive mice, across all groups there was severe gut microbiota dysbiosis and substantially reduced the bacterial diversity (P < 0.00001) (Fig. 6). Of note, *Lachnospiraceae* and *Bacteroides* populations were nearly eliminated and *Akkermansia* remained undetectable throughout the study. But, simultaneously extraordinary increase in abundance of *Enterobacteriaceae* (> 68.5%, P < 0.001) and *Enterococcaceae* (> 8.06%) populations were also recorded. These post-antibiotic events in the GI tract were found to be favorable for colonization by *P. aeruginosa* in healthy mice. However, after oral fucoidan supplementation in mice, by p.i. day 3, remarkable expansion in abundance of *Bacteroides* (29–50% RA, P < 0.001) from previously undetectable levels eventually replaced *Enterobacteriaceae* members as the most abundant genus (< 25% RA). In contrast, *Parabacteroides* was the predominant genus that mediated recovery from dysbiosis from p.i. day 3 to day 14 in the untreated group (Additional file 19: Fig. S10).

Notably, at p.i. day 7, *Bacteroides* and *Parabacteroides* were the two most prevalent genera (54% RA, P < 0.001) in the FV supplemented group, while in the AN supplemented group *Bacteroides* and *Lachnospiraceae*_NK4A136_group (61% RA, P < 0.001) were most prevalent. Consequently, *Enterobacteriaceae* was nearly eliminated in AN supplemented group (< 1% RA, P < 0.001) and strongly reduced in FV group (< 7% RA, P < 0.001) when compared to untreated group (< 15% RA). In the fecal samples from p.i. day 14 and day 30, no significant changes at genus level in % relative abundance were noted in both AN and FV treatment groups, indicating stable maintenance of established conventional community members for at least 2 weeks after discontinuation of fucoidan supplementation (Additional file 19: Fig. S10).

At the phylum level in the healthy control mice, *Firmicutes* (39.21%) and *Bacteroidetes* (58.87%) were predominant phyla in the fecal microbiota, with a combined relative abundance of 98.08% ± 0.21 and the F:B ratio ranged between 0.5 and 0.82 (Fig. 5B). After clindamycin treatment, % RA of *Firmicutes* decreased by 50% to 18.31% and *Bacteroidetes* were nearly eliminated (RA was 0.042%, P < 0.001). But, at the same time %RA of *Proteobacteria* remarkably increased to 81.62% from 0.46% (P < 0.001). Relative abundance of *Firmicutes* in the non-supplemented groups increased to ≥ 40% earlier (p.i. day 3) than in fucoidans supplemented groups (p.i. day 7–14) (Additional file 20: Fig. S11). Notably, *Bacteroidetes* abundances increased substantially and peaked earlier in fucoidans supplemented infected groups (day 3 for FA; day 7 for FV, P < 0.001) than in non-supplemented infected group (day 14). On p.i. day 30 in all the infected groups relative abundance of *Firmicutes* (61.35%) was higher than *Bacteroidetes* (37.45%), thereby increasing the F:B ratio to 1.67 ± 0.25, this ratio is higher than in healthy control mice (0.5–0.82) (Fig. 5B).

PCoA revealed that the healthy baseline microbial composition formed a cluster significantly (that is distinctly) separated from clindamycin treated and fucoidan supplemented mice (Fig. 6B). Among the *P. aeruginosa* infected groups at p.i. day 7 samples from fucoidans supplemented groups and non-supplemented control group clustered separately.

## Discussion

In *P. aeruginosa* and other gram-negative bacterial pathogens primary function of surface anchored proteins similar to TpsA1/CdiA2 and TpsA2/CdiA1 were found to promote bacterial competition through contact-dependent growth inhibition systems (CDI) [32]. However, in this study proteomic analysis revealed the presence of TpsA1 and TpsA2 in the culture supernatants. Additionally, when *P. aeruginosa* N-terminal TPS domain (1–360 aa) of TpsA1/TpsA2 (TpsA-NT) and its cognate transporter TpsB were expressed in *E. coli*, TpsA-NT (31 kDa) protein fragment was secreted into the culture media abundantly, suggesting that in addition to contact-dependent growth inhibition and/or contact-dependent growth signaling, secreted CdiA proteins might play a role in adhesion and colonization. Due to high homology (> 99% identity) at the N-terminal amino acid sequences (1–360 aa) between TpsA1 and TpsA2 proteins, secretion could be mediated either by TpsB1 or TpsB2. Homology between TpsA4 (LepA) and TpsA6 (CupB5) is around 56% at the N-terminus, but Garnett et al., showed that both the proteins could be translocated by TpsB4 (LepB), a cognate partner TpsA4 (LepA) [43]. Amino acid sequence alignment also revealed that, both *P. aeruginosa* and *E. coli* TpsA proteins contain all three conserved motifs necessary for secretion, therefore, *E. coli* protein expression system is suitable for expression of *P. aeruginosa* TpsA1 and TpsA2 proteins. Studies on structural and functional analysis of recombinant TpsA domains expressed in *P. aeruginosa* and *E. coli* might demonstrate similar results. Previously, to determine the structure of a *H. influenzae* TpsA protein secretion domain, HxuA_301_ (TpsA) and HxuB (TpsB) proteins were expressed successfully in *E. coli* strain BL21(DE3) [46].

Over the years, the N-terminal TPS domains of varying lengths (around 300 aa) were found to be both necessary and sufficient for secretion of FHA, HMW1 and ShlA by their cognate TpsB transporters [42, 47, 48]. In FHA like proteins TpsA1 & TpsA2 play a role in CDI with conserved N-terminal domain anchored to the bacterial surface and variable C-terminal CdiA effector proteins that are exposed [42]. In contrast, evidence from HMW1-like proteins TpsA3 (CdrA) suggested N-terminal end could be translocated across the outer membrane [48, 49].

Although bioinformatics tools for protein structure and function analysis tend to predict the presence of conserved HA domain at the start of N terminus (between 30 and 200 aa) of TpsA proteins in gram-negative pathogens [50, 51], there is no experimental evidence to support the role of hemagglutinin or lectin function, at least in proteins similar to FHA-NT. In this study we found that purified recombinant TpsA-NT-HA domain can bind to PGM that contains multiple HBGAs (ABH/Lewis type). When fucosylated glycans present on PGM were blocked with fucose specific lectins, TpsA-NT-HA domain interactions to PGM was inhibited only by LTL suggesting that both TpsA-NT and LTL can specifically bind to fucosylated glycans such as Lewis-X, Lewis-Y and 6’sulfo sialyl Lewis-X or share some glycan epitopes present on HBGAs [44]. Among the HBGAs tested, we found that TpsA-NT-HAD could consistently bind to and was inhibited by Lewis-B only with low affinity (471 μg/mL). It is possible that valency, spacing and orientation of the commercial HBGAs used for screening are different from the HBGAs present on PGM or natural interactions between TpsA-NT-HAD and PGM is multivalent i.e. TpsA-NT-HAD can bind to multiple HBGAs present on PGM simultaneously [52, 53].

Several human enteric pathogens bind to fucosylated human histo-blood group antigens (HBGAs) expressed on the gut mucosa, including *Campylobacter jejuni*, Norwalk virus and *Helicobacter pylori* [54, 55]. Mucins like, PGM, which contains multiple fucosylated, HBGAs (type A, H1, and Lewis b antigens) was found to bind multiple norovirus strains due to lectin-like recognition by the VP1 capsid protein [54]. The *H. pylori* adhesins BabA and SabA bind to mucin HBGAs, Lewis B and sialyl-Lewis A and sialyl-Lewis X, respectively [55]. By targeting lectin LecB, which can bind to fucosylated oligosaccharides, A, B, H, Lewis-A and Lewis-X blood, with multivalent fucosyl dendrimers which have 100-fold higher affinity toward LecB than fucose, complete inhibition of biofilm formation and dispersion was observed in *P. aeruginosa* isolates [56].

Like LecB, interactions of a secreted TpsA-NT-HA activity with HBGAs on host cell surface glycoproteins or mucins could play a crucial role in which might benefit *P. aeruginosa* to colonize the ultimate host tissue niche (lung, intestine and skin). By inhibition assays fucoidans (α-1,3-Fuc and α-1,4-Fuc sulfated polysaccharides) were found to be potent inhibitors of TpsA-NT interaction with mucin, this could be due to the presence of some structural motifs that could mimic predominantly sialyl Lewis-X, 6’sulfo sialyl Lewis-X and sialyl Lewis-A, suggesting that interaction between TpsA-NT and fucoidans is due to the molecular mimicry of HBGA glycans like sialyl Lewis-X. Several studies have shown that sulfated polysaccharides such as fucoidans, dextran sulfate, heparin and heparan sulfate, or some of their derivatives were able to prevent P-selectin binding to sialyl Lewis-X [57, 58].

Since dextran sulfates were the most potent inhibitors of T5SS-NT-HAD interaction to PGM it is tempting to speculate the use of DSS oral formulations against *P. aeruginosa* GI tract infections. However oral administration of DSS (36–50 kDa, 2–10%) induces colitis in laboratory animal models and mimics human ulcerative colitis pathology [59]. Currently we can only speculate that oral administration of high molecular weight DSS in non-colitogenic doses (0.01–0.1%) might be sufficient to neutralize T5SS-NT-HAD and other the virulence factors. Furthermore, desulfation and depolymerization of fucoidans and dextrans reduced the inhibitory potency, this indicates that high potency of fucoidans when compared to gastric mucins might be due to two mechanisms (1) molecular mimicry of blood group glycotopes by fucoidans and (2) interaction between positively charged residues present in TpsA-NT-HA with sulfate groups present in sulfated polysaccharides.

Preliminary studies on *B. pertussis* FHA indicated that the sulfated polysaccharides (heparin, dextran sulfate, and fucoidan) were able to inhibit *B. pertussis* FHA binding to immobilized heparin and attachment of CHO cells to FHA and predicted that specific glycosaminoglycan binding domain was located near the N-terminus of the FHA protein [60]. In our adhesion assay conditions, pre-incubation of *P. aeruginosa* with fucoidans reduced adhesion to Caco 2 cells. However, due to cytotoxic nature of clinical isolates and short interaction time between *P. aeruginosa* and epithelial cells, expression and/or secretion of TpsA may not be sufficient to see dramatic differences with fucoidan treatment. It is possible that along with TpsA1 and TpsA2, interactions of other unknown fucose-specific adhesins with glycoproteins are inhibited by fucoidans in the mammalian gastrointestinal tract. Although LecB can bind to host fucosylated blood groups glycans, we found that fucoidans used in this study did not inhibit the LecB binding to porcine gastric mucin, therefore we rule out the possibility of fucoidans inhibiting LecB binding to intestinal cells. Since the negative charge of the fucoidans results from the presence of sulfate residues in the C-2 and C-4 positions, occasionally in C-3, it might also function non-specifically by forming complexes with other positively charged amino acid residues present on adhesins of *P. aeruginosa* [61]. Further experiments are needed to examine potential interactions between fucoidans and flagella and type 4 pili, which are the other major adhesins of *P. aeruginosa*, capable of binding to host epithelial gangliosides, asialoGM1 and asialoGM2 [62].

Although alginates did not show high potency like dextran sulfates or fucoidans, among the subset of *P. aeruginosa* strains, alginate is a major component of extracellular polysaccharide matrix [63], even low affinity T5SS-NT-HAD interactions with alginate may play a role in neighboring cell–cell interactions and biofilm development. Our findings indicated dual functionality of TpsA-NT: (1) TPS domain on TpsA is targeted to TpsB and truly secreted onto the bacterial surface or into the surrounding culture medium; and (2) Subsequently, hitherto unknown TpsA-NT lectin like activity may mediate binding to multiple HBGAs present on mucins, glycosaminoglycans and sulfated polysaccharides like fucoidans. Currently, we can only speculate that multidomain TpsA1/TpsA2 proteins could confer intercellular binding by a two‐way handshake interactions through different domains, TpsA1/TpsA2 proteins that are released bind to mucins with N-terminus but the other C-terminal domains could still interact with TpsA1/TpsA2 proteins domains that remain anchored to the bacterial cell surface. In *P. aeruginosa*, TPS system, which encodes secreted adhesin CdrA was shown to promote auto-aggregation in liquid culture and biofilm formation using CdrA-Psl and CdrA-CdrA interactions [49]. Ag43 (Antigen 43), a type Va auto-transporter from *E. coli* utilizes self-recognizing handshake interaction mechanism to flocculate, which is beneficial in colonization, immune evasion and persistence in the host [64].

Since fucoidans are widely used in normal diets in East Asian countries and increasingly utilized as an ingredient in some dietary supplement products, we hypothesized that by using nutritional grade fucoidans, we may reduce *P. aeruginosa* colonization in GI tract. We observed that over time proportion of the mice that were decolonized further increased to 60%, while in the mice with persistent colonization, bacterial loads decreased significantly; on the other hand, decolonization at the end of the study period was found in only < 15% of the untreated mice.

*P. aeruginosa* overcomes the resistance to colonization mediated by gut microbiota and innate immune system [5, 65]. We also observed dramatic changes in gut microbiota. Intestinal microbiota was lower in richness and diversity after clindamycin treatment, compared to healthy controls. This post-antibiotic event, which was essential to facilitate *P. aeruginosa* colonization of the gut, suggests that members of *Bacteroidetes* and *Firmicutes* phyla promote resistance to colonization by *P. aeruginosa*, while the abundance of *Proteobacteria* enables pathogenic species colonization.

Among the fucoidan-supplemented mice, in the first week of decolonization *Bacteroides* was the most abundant genus, followed by *Lachnospiraceae*_NK4A136_group and *Parabacteroides*. In contrast in the control group, *Parabacteroides* was the predominant genus. After the withdrawal of fucoidan supplementation on day 14, between day 15 and day 30, stability at genus level was established in all groups mainly by *Bacteroides, Lachnospiraceae*_NK4A136_group and *Parabacteroides*; however, no significant decolonization was observed in the control group, suggesting a prophylactic role for prebiotic fucoidans in the treated groups. Overall, these important findings indicated at least two mechanisms by which prebiotic fucoidans could combat the colonization of *P. aeruginosa* in the mouse model, one involving inhibition of secreted virulence factors (TpsA/CdiA) interactions with mucins and the other by enriching the growth of *Bacteroides*.

Our study observations are consistent with earlier studies which reported that among the clindamycin-pretreated mice, *Bacteroides* were nearly eliminated or remained undetectable, *Enterobacteriaceae* proportions increased significantly while the reduction in *Lactobacillus* proportion was modest [66, 67]. Although at lower proportions, the continued presence of members of *Firmicutes* in clindamycin-pretreated mice may partially explain that for recovery from dysbiosis, members of *Firmicutes* play a major role among the fucoidan non-supplemented mice. Studies on impact of clindamycin treatment on humans also demonstrated that members of the *Bacteroides* species in the fecal microbiota remained reduced for 2 years following clindamycin therapy [68].

Studies on effectiveness of fucoidans to restore gut microbiota composition after antibiotic treatment are limited. In a mouse model of antibiotic-associated diarrhea (AAD), a recent study reported that dietary sulfated polysaccharide isolated from *Gelidium pacificum* Okamura (GPOP-1) restored the gut microbiota composition by significantly increasing the abundance of *Bacteroides*, *Oscillospira*, *Bifidobacterium* and decreasing the abundance of *Parabacteroides*, *Sutterella*, AF12. Furthermore, GPOP-1 improved mucosal barrier function, downregulated inflammatory cytokines and increased the production of SCFAs [69]. In another study, in mice treated with ciprofloxacin-metronidazole, dietary *Ascophyllum nodosum* improved colonic health and partially restored the dysbiosis of gut microbiota by increasing the abundance of beneficial bacteria (*Ruminococcaceae*_UCG_014 and *Akkermansia*) and decreasing the abundance of harmful bacteria (*Proteus* and *Enterocococus*) [70]. Of note, *Akkermansia* spp. (associated with human health) had no role in decreasing the *P. aeruginosa* colonization and fucoidans cannot restore *Akkermansia* spp. once eliminated from the GI tract by clindamycin [71].

In general, bacteria colonizing the intestinal epithelium are exposed to highly sulfated glycans present on mucins, glycosaminoglycans and sulfated polysaccharides (fucoidans) used in diets, which are potential carbon and nitrogen sources for cell proliferation and metabolite biosynthesis, but their high sulfate content could prevent their degradation [72–74]. Specialized organisms like *Bacteroides* spp. containing both glycoside hydrolases and sulfatases could effectively depolymerize and utilize complex carbohydrates for metabolism and to release monosaccharides into the gut lumen for other organisms to scavenge [72, 73]. Previously, Benjdia et al. demonstrated that deletion of *Bacteroides thetaiotaomicron* genes encoding for anaerobic sulfatase-maturating enzyme (anSME) (BT0238) resulted in loss of sulfatase activity and impaired ability to use sulfated glycosaminoglycans (chondroitin and heparin) as carbon source [72]. Recently Sato et al. showed that most *Bacteroides* species could degrade and assimilate sulfated GAGs and mucin that are available to the gut microbiota and secreted essential amino acids, γ-amino butyrate (GABA) and short-chain fatty acids which are needed for human health [75]. Based on the results of this study, we can hypothesize that, among the fucoidans supplemented mice, *Bacteroides spp.* may effectively depolymerize fucoidans to fucose as carbon and nitrogen source. Consequently, selectively promote the growth of members of genus *Bacteroides* to occupy the niche space that overlaps with *P. aeruginosa.*

Finally, despite being housed in a single cage fucoidans treated mice that were once decolonized could not be recolonized by *P. aeruginosa*, suggesting fucoidans may also limit the transmission of pathogens through fecal to oral route. In a mouse infection model Bachta et al. showed that *P. aeruginosa* can spread from the bloodstream to the gallbladder where it could multiply to high numbers, and subsequently to the intestines and feces, thereby allowing transmission of *P. aeruginosa* to uninfected animals [24]. We therefore assume that prophylactic supplementation of fucoidans might be beneficial to some patients in the clinical settings, as a recent study in the US observed that in the ICU setting the piperacillin-tazobactam treatment contributed to microbiota disruption characterized by loss of diversity and protective taxa, and subsequent carbapenem-resistant *P. aeruginosa* colonization [5]. Burke et al. further reported that cystic fibrosis patients who have been treated with ≥ 5 courses of intravenous antibiotics per year had the lowest *Bacteroidetes* and highest *Firmicutes* proportions in the gut [76].

There are limitations of this study. Fucose utilization by intestinal commensal like *Bacteroides* was not analyzed and the level of fecal short-chain fatty acids (SCFAs) such as acetate, propionate and butyrate were not evaluated in the fucoidan-supplemented mice [19, 77]. Although we did not evaluate the role of metabolites produced by fucoidans enriched microbiota in improving the gut homeostasis, emerging evidence tends to support the hypothesis that *Bacteroides* and *Bacteroides-*derived propionate is beneficial to individuals with cystic fibrosis [78]. By using an in vitro fermentation model Liu et al. demonstrated that among the individuals with strong utilization of sulfated polysaccharides, gut microbiota possessed either more *Parabacteroides* or more *Bacteroides*, consequently more beneficial metabolites [79].

## Conclusions

Fucose-rich glycans-targeting adhesins may play a major role in mediating *P. aeruginosa* attachment to mucins in gastrointestinal tract, which is evident by the robust decolonization effect of marine prebiotic fucoidans. We demonstrated two mechanisms by which fucoidans mediate their protective effects: first, inhibition of virulence factors (TpsA/CdiA) interactions with mucins and second, selectively promoting the growth of beneficial *Bacteroides* species. Further studies are needed to examine the clinical application of fucoidans to prevent *P. aeruginosa* colonization and infection.

## Supplementary Information

**Additional file 1.** Methods.

**Additional file 2: Table S1.** Strains used in this study are listed below.

**Additional file 3: Table S2.** Two-partner secretion (TPS) family TpsA proteins in culture supernatants identified by LC–MS/MS proteomic analysis.

**Additional file 4: Table S3.** Primers used in this study are listed below.

**Additional file 5: Table S4.** Polysaccharides used in this study.

**Additional file 6: Table S5.** Antibodies used in this study.

**Additional file 7: Table S6.** Lectins used in this study.

**Additional file 8: Fig. S1.** Haemagglutinin domains (HAD) present at N-terminus of TpsA, Type V secretion system proteins in *Pseudomonas aeruginosa*. Six different TpsA proteins can be found in P. aeruginosa PA14 strain. Haemagglutinin domains (HAD) are very well conserved in all the T5SS proteins. Both Tps1 and Tps2 protein sequences can be found in genomes of > 80 strains in Pseudomonas Genome Database (PGDB). Images were generated by BLAST program http://blast.ncbi.nlm.nih.gov/Blast.cgi.

**Additional file 9: Figure S2.** Preliminary screening for interaction of TpsA-NT-HAD with different substrates coated on the microliter plate. Porcine gastric mucin type III (Sigma) showed high reactivity in the ELISA assay. Alfa glycoprotein (AGP), human ovarian cyst mucin (HOC), bovine submaxillary mucin (BSM), pig submaxillary mucin (PSM). Numbers indicate fractions. Porcine gastric mucin #4, a blood group A + H substance, that was derived from crude Porcine stomach mucin. Treatment of mucin #4 with HCl (pH 2.0, 90 min, 100 °C) yields Porcine gastric mucin #9, while acid hydrolysis (pH 1.5, 100 °C, 2 and 5 h) gives Porcine gastric mucins #14 and #21, respectively [12].

**Additional file 10: Table S7.** IC50 values of polysaccharides to inhibit LecB-PA01 monomer from binding to Porcine Gastric Mucin Type III obtained by ELISA.

**Additional file 11: Figure S3.** Binding of blood group antigen-specific antibodies to porcine gastric mucin type III (PGM), *Fucus vesiculosus* fucoidan (FV) and *Fucus serratus* fucoidan (FS)*.* TpsA-NT-HAD indicates N-terminal hemagglutinin domain in TpsA1 and TpsA2 proteins. Representative results for interaction between antibodies and immobilized PGM, FV and FS. A color gradient heat map, with high reactivity (Red) to no reactivity (Blue) based on lectin ELISA OD values, has been applied to the well values. Antibodies used in this study are shown in Additional file 6: Table S5.

**Additional file 12: Figure S4.** Binding of specific plant lectins fucoidans, *Fucus vesiculosus* crude (FVC), *Fucus vesiculosus* 95% (FVP), *Fucus serratus* fucoidan (FS) and *Ascophyllum nodusum* (AN). Previously well-characterized porcine gastric mucin type III (PGM) was used as s positive control. Representative results for interaction between plant lectins and immobilized PGM, FVC, FVP, FS and AN. A color gradient heat map, with high reactivity (Red) to no reactivity (Blue) based on lectin ELISA OD values, has been applied to the well values. Note: Dilutions of lectins AAL and ConA in buffer were fourfold. Reactivity was low with other lectins including, SNA, PNA, GNA, DFL, WGA, SBA, DBA. Carbohydrate specificities of major plant lectins used in this study are shown in Additional file 7: Table S6.

**Additional file 13: Figure S5. Inhibition of** *P. aeruginosa* S8 adhesion to Caco-2 intestinal cells **by** *Fucus vesiculosus* fucoidan (FV) (A) and *Fucus serratus* fucoidan (FS) (B). *P. aeruginosa* S8 were pre-incubated with fucoidans in 250-μl DMEM medium for 1 h at 37 °C in 5% CO_2_ and added to monolayers of Caco-2 cells with an MOI of 10 (2 X 10^5^ cells) and incubated for additional 1 h at 37 °C in 5% CO_2._ Significant differences using Chi Square test in adhesion in comparison to the adhesion of bacteria incubated without fucoidan are indicated by asterisk (**, *P* ≤ 0.01; ***, *P* ≤ 0.001). MOI: Multiplicity of infection (Bacterium: host cell).

**Additional file 14: Figure S6. Inhibition** of bacterial adhesion to Caco-2 intestinal cells in the comparison of fucoidans with monosaccharides**.** *P. aeruginosa* S8 (A) and ESBL-producing ST131 *E. coli* as well as Non-ESBL-producing ST131 *E. coli* (B) were tested. Each test was pre-incubated with for 1 h at 37 °C in 5% CO_2_ and added to monolayers of Caco-2 cells (2 X 10^5^ cells) and incubated for additional 1 h at 37 °C in 5% CO_2_. Fucoidans (FV, *Fucus vesiculosus*; FS, *Fucus serratus*; LJ, *Laminaria Japonicia*) were used (each 12.5 mg/mL)*.* Monosaccharides (10 mM), including dextran (Dex), mannose (Man), glucose (Glc), D-fucose (D-F), were tested. Significant difference in inhibition of bacterial adhesion with fucoidan treatment compared to the adhesion without fucoidan treatment is indicated by asterisk (**, *P* ≤ 0.01; ***, *P* ≤ 0.001). ESBL: extended-spectrum β-lactamase; ns: no significance.

**Additional file 15: Figure S7.** Sulfate content of fucoidan samples after thermal treatment (T) at 80 °C. A, 10 and B, 60 min; and after C, overnight acid hydrolysis at 80 °C to liberate sulfate ions in thermally treated samples. HMW-untreated high molecular weight fucoidan Fv (*Fucus vesiculosus* 95%) as control. T-purified – thermally treated fucoidan Fv, its filtrate and mixture of both (mix). T10 and T60- purified Fv (*Fucus vesiculosus* 95%) obtained after 10 or 60 min at 80 °C, purified and hydrolyzed in HCl overnight at 80 °C to release sulfate ions.

**Additional file 16: Figure S8.** Fucose content of fucoidan Fv (*Fucus vesiculosus* 95%) samples after thermal (T) and thermal treatment and acid (T + A) treatment at 80 °C. A,D 10 and B,E 60 min; and after C,F overnight acid hydrolysis at 80 °C to liberate sulfate ions and sugar monomers. HMW-untreated high molecular weight fucoidan Fv (*Fucus vesiculosus* 95%) as control.

**Additional file 17: Table S8.** Significant reductions in fecal *P. aeruginosa* load among the study groups during the study period.

**Additional file 18: Figure S9.** Decolonization and recolonization in individual mice after fucoidan-treatment. Time lines of fecal shedding tests were scheduled for 30 days, including 14 days treatment of fucoidans Fv (*Fucus vesiculosus*) and F_A_ (*Ascophyllum nodusum*) after challenge of Shanghai-fever strain *P. aeruginosa* S8. Negative control (NC) was the mouse challenged with water alone. Oral challenge of *P. aeruginosa* (1.0 × 10^7^ CFU) was applied for three days from Day-2 to Day 0. Fucoidans FVF, and ANF (0.5% in water, w/v) were used to feed mice (10 mice per each group) from Day-4 to Day + 14.

**Additional file 19: Figure S10.** Changes in relative abundance at genus level were compared between groups on post infection (P.I.) day 3, 7, 14 and 30. Differences between groups were calculated using a t-test, numbers above columns indicate *P* values (significant difference, *P* < 0.05).

**Additional file 20: Figure S11.** Changes in relative abundance at phylum level were compared between groups on post infection (P.I.) day 3, 7, 14 and 30. Differences between groups were calculated using a t-test, numbers above columns indicate *P* values (significant difference, *P* < 0.05).

## Data Availability

Data are available upon request. The majority of results from this study are included in the article or uploaded as supplementary figures and tables.
