# Supplementary material for: Marine prebiotics mediate decolonization of Pseudomonas aeruginosa from gut by inhibiting secreted virulence factor interactions with mucins and enriching Bacteroides population
Source: J Biomed Sci. 2023 Feb 2;30:9. doi: 10.1186/s12929-023-00902-w (PMC9896862; doi:10.1186/s12929-023-00902-w)
Supplement: Supplementary file 1 — Additional file 1. Methods. [file 12929_2023_902_MOESM1_ESM.docx]

**Additional file 1: Supplementary Methods**

**Protein expression and purification**

PCR fragments containing the TpsA-N-terminal haemagglutinin (HA) domains (TpsA-NT-HAD, 49-332aa, 29 kDa) coding sequences were amplified using genomic DNA from *P. aeruginosa* (PA01 or PA14) and primers PA14-00510-145bp -NdeI F (5’ AGATATACATATGGGCGTGCCCATCGTCAATA 3’) and PA14-00510-995bp –HindIII (5’ TATGCTAAGCTTGCTCTGCCGGTTGACCAGT 3’) (Additional file 4: Table S3) [1,2]. Amplified fragments were digested with NdeI and HindIII and ligated to pET29b+ (Novagen, Madison, WI, USA), with the resulting plasmids transformed into *E. coli* DH5α (Fig. 1; Additional file 4: Table S3). Recombinant protein TpsA-NT-HAD was expressed in *E. coli* BL21 (DE3) (RBC Bioscience, Taipei, Taiwan) after induction with 1mM IPTG and purified according to the manufacturer’s instructions for Ni2+ affinity chromatography using Nickel-Chelating Resin (Ni Sepharose 6 Fast Flow, GE Healthcare, Uppsala, Sweden). Fucose specific lectin LecB monomer was expressed similarly in *E. coli* (Additional file 4: Table S3)*.* Protein preparations were evaluated by sodium dodecyl sulfate polyacrylamide gel electrophoresis (SDS–PAGE) and Coomassie blue staining or Western blotting. Purity of >95% was observed for all the proteins after Coomassie-staining of gels. TpsA-NT-HAD protein that was used in ELISA assays migrated as a 31kDa band, consistent with its estimated molecular weight of 30.65kDa after codon optimization and histidine tag addition (<https://web.expasy.org/compute_pi/>).

For analysis of the minimal region essential for secretion of TpsA1 and TpsA2 proteins, PCR fragments containing the *P. aeruginosa* TpsA-N-terminal protein domain (TpsA-NT31, 29-332 amino acids, 31kDa) coding sequences were amplified using primers 160F (starting from TpsB) and 161R (ending with TpsA) digested with EcoRV and Sac1 and ligated to pET29b+. Since TpsB is essential for TpsA secretion, both proteins TpsB (full length) and TpsA-NT (31 kDa) were co-transcribed from plasmid pET29b+. Recombinant protein TpsA-NT31 was expressed in *E. coli* BL21 (DE3) (RBC Bioscience, Taipei, Taiwan) after induction with 1mM IPTG. Next, culture supernatants (1 ml) collected after induction with IPTG were precipitated with acetone after overnight incubation at -80°C [3], while the culture pellet was lysed by heating for 10 minutes at 95°C protein. Protein preparations were evaluated by SDS–PAGE and Coomassie blue staining. TpsA-NT31 in culture supernatants migrated as a 32kDa band after standard Western blotting immunodetection with anti-histidine6 tag rabbit IgG (1:10000, Bioman, Taiwan) and goat anti-rabbit IgG HRP-conjugated (1:10000, Millipore, USA). This was consistent with its estimated molecular weight of 32.76kDa after codon optimization and histidine tag addition.

Furthermore, although both the fragments TpsA-NT15 (31-180aa residues, 15kDa) and TpsA-NT31 (31-332aa residues, 31kDa) were expressed in *E. coli* (confirmed by Western blot), we could not purify the fragments, probably due to rapid cytoplasmic degradation during the purification process.

**Identification fucosylated histo-blood group antigen (HBGA) and fucosylated glycans on porcine gastric mucin or fucoidans**

To identify and characterize fucosylated histo-blood group antigen (HBGA) and fucosylated glycans on porcine gastric mucin or fucoidans, substrates PGM (1-2 μg) and fucoidans (100 μg) in 100 μL TSB were used to coat a 96-well microtiter plate (Costar, USA) for 18 hours at 4°C and blocked with TSMBB for 1 hour at room temperature (22°C). The micro wells were filled with 100 μL serially diluted anti-HBGA monoclonal antibody (4 μg to 0.04 μg or 1:20 to 1:2560) or biotinlyated lectin (6 μg to 0.04 μg) and incubated for 1 hour at room temperature (22°C). Blood group antigen antibodies used were mostly IgM antibodies that are commercially available and were well characterized previously, either by manufacturers or research groups (Additional files 6 and 7: Tables S5 and S6) [4]. After washing the assay plate three times with TSMWB, wells were incubated with 100 μL horseradish peroxidase conjugated-secondary monoclonal antibody (1:5000, Jackson ImmunoResearch Inc. USA) or horseradish peroxidase conjugated-streptavidin (1:1000, R&D, Systems, USA) for 1 hour at room temperature (22°C). Plates were developed by adding 100 μL substrate, tetramethylbenzidine (NeA-Blue, Clinical Science Products, USA) and the change in absorbance after 30 minutes was determined at 650 nm by SpectraMax M5 plate reader (Molecular Devices, USA) or after stopping the reaction by 50 μL 1N sulfuric acid, the change in absorbance at 450 nm was noted. Buffers used in ELISA assays were prepared as described earlier by Heimburg-Molinaro et al. [5].

TS Buffer (TSB) = 20mM Tris-HCl, pH 7.4 150mM NaCl

TSM Buffer (TSMB) = 20mM Tris-HCl, pH 7.4 150mM NaCl, 2mM CaCl2, 2mM MgCl2

TSM Wash Buffer (TSMWB) = TSM Buffer + 0.05% Tween-20

TSM Binding Buffer (TSMBB) = TSM buffer + 0.05% Tween-20 + 1% BSA

TSB+ 0.05% Tween-20 + 1% BSA

**Preparation of low molecular weight fucoidan by mild acid hydrolysis**

To obtain low molecular weight (LMW) fucoidan, 100 μL solution of fucoidan *Fucus vesiculosus* (Sigma, purity >95%), high molecular weight (HMW), in bidistilled water (10mg/mL) was mixed with 0.2 M HCl in a 1:1 ratio and the mixtures were heated for different time intervals (10 minutes - 1 hour) at 80°C, then neutralized with 1M Tris-HCl pH=7.5 [6]. The hydrolysates were fractionated and concentrated by ultracentrifugal Amicon Ultra 0.5 filters (molecular weight cutoff: 10 kDa, Merck Millipore), washed 2x with 450 μL of water at 130rpm for 10min and evaporated to dryness by SpeedVac for 2h at room temperature. To detect sulfated polysaccharide and estimate their size distribution carbohydrate polyacrylamide gel electrophoresis (C-PAGE) was used. Fucoidans and their fractions in polyacrylamide gels were visualized by silver, alcian blue (data not shown) and o-toluidine blue staining [7]. 0.1% o-toluidine blue in 2 % acetic acid was used to stain the gels for 1 hour and distained by 2 % acetic acid 2x 30 min. Other polysaccharides *Fucus serratus* (Fs), dextran-sulfates (DS 500kDa), chondroitin sulfate A sodium salt 10-30kDa (CS), hyaluronan 8-15kDa (HA), alginates (marine *A. nodosum* and *L. japonica* and bacterial *P. aeruginosa* and *A. vinelandi*) and heparin sodium salt 10-12kDa (HS) were used as positive and negative controls for size distribution.

To study the effect of mild acid hydrolysis on the fucoidan sulfation, sulfate analysis was performed using a sulfate assay kit (KA1621, Abnova, Taiwan). The method utilized the quantitative formation of insoluble barium sulfate in polyethylene glycol. The turbidity was measured between 560nm and was proportional to sulfate level in the samples. Fucoidan samples before and after mild acid hydrolysis were treated in 0.2M HCl at 80 °C overnight to liberate sulfate ions. Serially diluted sulfate standard was used to generate a calibration curve. Only after thermal treatment the sulfate content dropped by around 10% in 10 min treated fraction (T10’) and by 35% in 60 min treated fraction (T60’) compared to untreated high molecular weight fucoidan Fv (HMW). T10’ fucoidan fraction smeared band did not show loss in size on C-PAGE compared to untreated HMW Fv. Therefore, mild acid hydrolysis was applied to obtain low molecular weight fragments.

Fucose analysis was performed using l-fucose microplate assay procedure as described by the manufacturer (K-FUCOSE 04/18, Megazyme, Ireland). In this procedure, l-Fucose was oxidised by the enzyme l-fucose dehydrogenase in the presence of nicotinamide-adenine dinucleotide phosphate (NADP^+^) to l-fucono-1,5-lactone with the formation of reduced nicotinamide-adenine dinucleotide phosphate (NADPH). The amount of NADPH formed in this reaction was stoichiometric with the amount of l-fucose. It was the NADPH, which was measured by the increase in absorbance at 340 nm. Serially diluted l-fucose was used to generate a calibration curve. The total sugar content was also analyzed by the phenol-sulfuric acid method with L-fucose as the standard. Aqueous samples containing 2.5 wt % of the fucoidan were prepared, a set of fucose standards, at 30–150 mg/L, and relevant blanks. 1 mL of each solution (either calibration, water or sample) was placed into a 15 mL Pyrex tube with 4.5 mL of 6:1 v/v H_2_SO_4_ (98% purity). The tubes were capped, inverted several times to mix and left at room temperature for 5 min. Each tube was then placed in a boiling water bath for exactly 10 min, removed and cooled under tap water to quench the reaction. 0.1 mL of 3% aqueous cysteine hydrochloride was added to each tube, inverted several times and left for 30 min before measuring at 396 and 427 nm in a UV/VIS spectrophotometer [8].


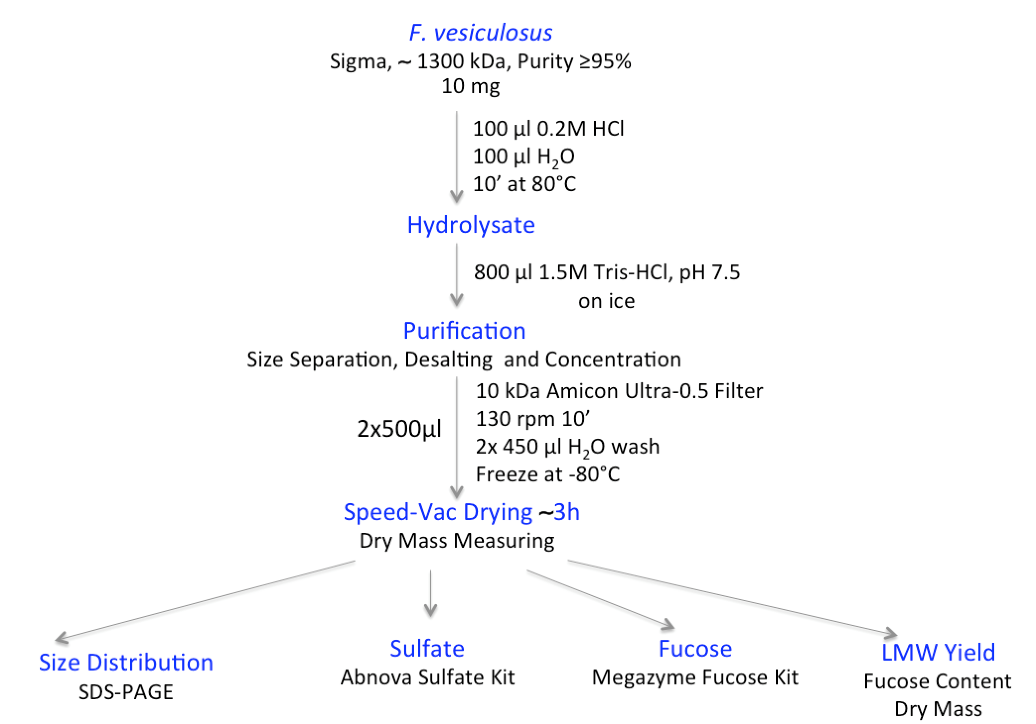


**Scheme of preparation of low molecular weight fucoidan by mild acid hydrolysis**

**Adhesion and inhibition assays**

In vitro cell adhesion and inhibition assays were performed using human cell lines (A549 and Caco-2 cells) [9,10]. A549 and Caco-2 cells were seeded into six-well plates at a density of 1.5×10^5^ cells/well. After 4 days of incubation and a cell density of 1.5×10^6^/well, bacteria were added with or without fucoidans to each well at an MOI of 10 & 100 and incubated for 1h at 37°C, non-adherent bacteria were eliminated by washing thrice with phosphate-buffered saline. Cells were lysed with 0.05% Triton X-100 solution for 10 minutes. Bacteria were counted by serial dilution and plating. All the cellular studies were performed in three independent experiments.

**16S rRNA sequencing and analysis**

### To investigate the role of fucoidans in bacterial dysbiosis, changes in microbiota profile due to virulent *P. aeruginosa* S8 colonization of the gut in mice were identified after analyzing the 16S rRNA sequencing data. Stool samples were collected from mice at several time points during the study period for 16S rRNA sequencing. Both sequencing and analysis were done at Genomic Medicine core laboratory, Chang Gung Memorial Hospital, Linkou. DNA was first extracted from stool specimens using the QIAamp PowerFecal^®^ DNA Kit (Qiagen, USA) according to the manufacturer's protocol. PCR and sequencing were performed as described earlier by Yeh et al [11]. Briefly, the 16S rRNA gene was amplified with region-specific primers that included the Illumina adaptor overhang nucleotide sequences (forward, 5′-TCGTCGGCAGCGTCAGATGTGTATAAGAGACAGCCTACGGGNGGCWGCAG-3′, and reverse, 5′ GTCTCGTGGGCTCGGAGATGTGTATAAGAGACAGGACTACHVGGGTATCTAATCC-3′). Subsequently, the product was subject to index PCR with Illumina® Nextera™ DNA UD Indexes. The final amplicon libraries were approximately 630 bp in length and were validated using the Agilent 2100 Bioanalyzer with Agilent HS DNA Kit. The sequencing of the multiplexed pooled libraries was carried out on the MiSeq System with MiSeq Reagent Kit v3 (600-cycle) (Illumina, USA). The sequencing reads were initially de-multiplexed using MiSeq Reporter v2.6, according to sample barcodes. Processing of raw reads was performed mainly using USEARCH v11 (<https://drive5.com/>) as described previously by Yeh et al. [11].

**References**

1. Holloway BW, Krishnapillai V, Morgan AF. Chromosomal genetics of Pseudomonas. Microbiol Rev. 1979 Mar;43(1):73-102.
2. Lee DG, Urbach JM, Wu G, Liberati NT, Feinbaum RL, Miyata S, Diggins LT, He J, Saucier M, Déziel E, Friedman L, Li L, Grills G, Montgomery K, Kucherlapati R, Rahme LG, Ausubel FM. Genomic analysis reveals that Pseudomonas aeruginosa virulence is combinatorial. Genome Biol. 2006;7(10):R90.
3. Crowell AM, Wall MJ, Doucette AA. Maximizing recovery of water-soluble proteins through acetone precipitation. Anal Chim Acta. 2013;796:48‒54.
4. Mitsuoka C, Kawakami-Kimura N, Kasugai-Sawada M, Hiraiwa N, Toda K, Ishida H, Kiso M, Hasegawa A, Kannagi R. Sulfated sialyl Lewis X, the putative L-selectin ligand, detected on endothelial cells of high endothelial venules by a distinct set of anti-sialyl Lewis X antibodies. Biochem Biophys Res Commun. 1997 Jan 23;230(3):546-51. Erratum in: Biochem Biophys Res Commun 1997 Apr 17;233(2):576.
5. Heimburg-Molinaro J, Song X, Smith DF, Cummings RD. Preparation and

analysis of glycan microarrays. Curr Protoc Protein Sci. 2011;64:12.10.1–29.

1. Pomin VH, Valente AP, Pereira MS, Mourão PA. Mild acid hydrolysis of sulfated

fucans: a selective 2-desulfation reaction and an alternative approach for preparing

tailored sulfated oligosaccharides. Glycobiology. 2005;15(12):1376‒85.

1. Silchenko AS, Rasin AB, Kusaykin MI, Kalinovsky AI, Miansong Z, Changheng L, et al. Structure, enzymatic transformation, anticancer activity of fucoidan and sulphated fucooligosaccharides from *Sargassum horneri*. Carbohydr Polym. 2017;175:654‒60.
2. DuBois M, Gilles KA, Hamilton JK, Rebers PA, Smith F. Colorimetric method for determination of sugars and related substances. Anal Chem. 1956;28(3):350–6.
3. Chuang CH, Wang YH, Chang HJ, Chen HL, Huang YC, Lin TY, et al. Shanghai fever: a distinct *Pseudomonas* *aeruginosa* enteric disease. Gut. 2014;63(5):736‒43.
4. Chuang CH, Janapatla RP, Wang YH, Chang HJ, Huang YC, Lin TY, Chiu CH. *Pseudomonas* *aeruginosa*-associated diarrheal diseases in children. Pediatr Infect Dis J. 2017;36(12):1119‒23.
5. Yeh YM, Cheng HT, Le PH, Chen CC, Kuo CJ, Chen CL, et al. Implementation of fecal microbiota transplantation in a medical center for recurrent or refractory *Clostridioides difficile* infection and report of preliminary outcome. Biomed J. 2022;45(3):504‒11.
6. Wu AM, Wu JH, Singh T, Liu JH, Herp A. Lectinochemical studies on the affinity of Anguilla anguilla agglutinin for mammalian glycotopes. Life Sci. 2004 Jul 16;75(9):1085-103.
