# Supplementary material for: Marine prebiotics mediate decolonization of Pseudomonas aeruginosa from gut by inhibiting secreted virulence factor interactions with mucins and enriching Bacteroides population
Source: J Biomed Sci. 2023 Feb 2;30:9. doi: 10.1186/s12929-023-00902-w (PMC9896862; doi:10.1186/s12929-023-00902-w)
Supplement: Supplementary file 2 — Additional file 2: Table S1. Strains used in this study are listed below. [file 12929_2023_902_MOESM2_ESM.docx]

**Additional file 2: Table S1**

Strains used in this study are listed below

| **Strains** | **Relevant characteristics** | **Origin** |
| --- | --- | --- |
| *Pseudomonas aeruginosa* | |  |
| PAO1 | Infected burn/wound isolate | Lab collection [1] |
| PA14 | Infected burn/wound isolate | Lab collection [2] |
| PSA1/S1 | Shanghai fever isolates | Lab isolate [9,10] |
| PSA6/S6 |  |  |
| PSA8/S8 |  |  |
|  |  |  |
| *Escherichia coli* | |  |
| DH5α | *fhuA2* Δ(*argF-lac*)U169 *phoA* *glnV44* *gyrA96* *recA1 relA1 endA1 thi-1 hsdR17* [Φ80' lacZ(del)M15] | Lab collection |
| BL21(DE3) | *ompT* *gal* *dcm* *lon* *ΔhsdS* [λ(DE3)] | Lab collection |
