# Supplementary material for: Marine prebiotics mediate decolonization of Pseudomonas aeruginosa from gut by inhibiting secreted virulence factor interactions with mucins and enriching Bacteroides population
Source: J Biomed Sci. 2023 Feb 2;30:9. doi: 10.1186/s12929-023-00902-w (PMC9896862; doi:10.1186/s12929-023-00902-w)
Supplement: Supplementary file 3 — Additional file 3: Table S2. Two-partner secretion (TPS) family TpsA proteins in culture supernatants identified by LC–MS/MS proteomic analysis. [file 12929_2023_902_MOESM3_ESM.docx]

| **Additional file 3: Table S2**  Two-partner secretion (TPS) family TpsA proteins in culture supernatants identified by LC-MS/MS proteomic analysis. | | | | | | | | | | |
| --- | --- | --- | --- | --- | --- | --- | --- | --- | --- | --- |
| **Isolate secretome** | **TpsB - TpsA new locus_tag** | **TpsA Protein PA01 / PA14** | **Designated** | **mol. wt (Da)** | **unique peptides** | **unique spectra** | **Number of total spectra** | **% of total spectra** | **% sequence coverage** | **Protein identification probability** |
| PSA6 | PLES_RS14730 - PLES_RS14735 | PA2462- PA14_32790 | CdiA2 / TpsA1 | 574,982.00 | 10 | 11 | 63 | 0.06% | 31.60% | 100% |
|  | PA2463-  PA2462 | PA2462 - PA14_32790 | CdiA2 / TpsA1 | 573,166.00 | 145 | 174 | 1170 | 1.05% | 34.20% | 100% |
|  | PLES_RS00225-PLES_RS00230 | PA0041 - PA14_00510 | CdiA1/  TpsA2 | 361,764.10 | 32 | 36 | 126 | 0.11% | 36.50% | 100% |
|  | PA14_61190 - PA14_61200 | PA4625 - PA14_61200 | TpsA3 | 203,328.00 | 46 | 55 | 229 | 0.21% | 29.80% | 100% |
| PSA1 | PA14_32780 - PA14_32790 | PA2462- PA14_32790 | CdiA2 / TpsA1 | 574,982.00 | 24 | 26 | 51 | 0.04% | 5.72% | 100% |
|  | PA14_00490 PA14_00510 | PA0041 - PA14_00510 | CdiA1/  TpsA2 | 352,760.10 | 3 | 4 | 4 | 0.00% | 7.96% | 100% |
|  | PA4624-  PA4625 | PA4625 - PA14_61200 | TpsA3 | 219,741.50 | 33 | 37 | 108 | 0.09% | 22.00% | 100% |
|  | A0K_RS03800-A0K_RS03795 |  |  |  |  |  |  |  |  |  |
| PAO1 | PA2463-  PA2462 | PA2462- PA14_32790 | CdiA2 / TpsA1 | 573,166.00 | 112 | 121 | 529 | 0.46% | 27.10% | 100% |
|  | PA0040-  PA0041 | PA0041 - PA14_00510 | CdiA1/  TpsA2 | 361,582.30 | 17 | 19 | 56 | 0.05% | 25.50% | 100% |
|  | PA4624-  PA4625 | PA4625 - PA14_61200 | TpsA3 | 219,741.50 | 36 | 40 | 122 | 0.11% | 23.90% | 100% |

Note: Scaffold software (Proteome Software Inc.; version Scaffold-2_06_02) was used for validation of peptide & protein IDs, the alignment of samples and the quantitative comparison of samples based on spectral counting. Protein identification probability was generated by Scaffold software. Peptide Thresholds: 95.0% minimum. Protein Thresholds: 99.0% minimum and 1 peptides minimum.
