# Supplementary material for: Marine prebiotics mediate decolonization of Pseudomonas aeruginosa from gut by inhibiting secreted virulence factor interactions with mucins and enriching Bacteroides population
Source: J Biomed Sci. 2023 Feb 2;30:9. doi: 10.1186/s12929-023-00902-w (PMC9896862; doi:10.1186/s12929-023-00902-w)
Supplement: Supplementary file 4 — Additional file 4: Table S3. Primers used in this study are listed below. [file 12929_2023_902_MOESM4_ESM.docx]

**Additional file 4: Table S3.**

Primers used in this study are listed below.

| **Number** | **Primer Name** | **Primer Sequence** | **Ann.Temp. (C)** | **Size BP** | **Note** |
| --- | --- | --- | --- | --- | --- |
| 23F | PA14-00510 +145bp NdeI | 5’ AGATATACATATGGGCGTGCCCATCGTCAATA 3’ | 55 | 883 | For 30.65 kDa.  TpsA-NT protein purification.  Interaction Inhibition assays by ELISA.  Can express, can purify. |
| 24R | PA14-00510 +995bp HindIII | 5’ TATGCTAAGCTTGCTCTGCCGGTTGACCAGT 3’ |  |  |  |
|  |  |  |  |  |  |
| 160F | PA14-00490 +1 EcoRV | 5’ CCATGGCTGATATCGATGGCTCTCCATCGCCTGGCCTTTATC 3’ | 55 | 2833 | TpsA-NT31 domain protein (29-332 amino acids, 31kDa) For, 32.76 kDa  TpsA-NT31, TPS domain role in secretion, analysis by  Western blot |
| 161R | PA14-00510 +995 Sac1 | 5’ GTCGACGGAGCTCGACGCCAGGCTCTGCCGGTTGACCAGT 3’ |  |  |  |
|  |  |  |  |  |  |
| 169F | PA14-00510 +91bp NdeI F | 5’ AGATATACATATGCTGGCGCTGGACAAGGCCGCC 3’ | 55 | 477 | 17.69 kDa |
| 170R | PA14-00510R +540 EcorV | 5’ GAATCCGATATCGCGATGTCGCCGCCGTCCACCTGGAA 3’ |  |  |  |
| 171R | PA14-00510R +1584 EcorV | 5’ GAATCCGATATCGCCAGCAGGTCGTCGCCGATCAGCTT 3’ |  | 1527 | 53.89 kDa |
| 24R | PA14-00510 +995bpHindIII | 5’ TATGCTAAGCTTGCTCTGCCGGTTGACCAGT 3’ |  | 931 | 32.69 kDa |
|  |  |  |  |  | For, Protein purification  Interaction Inhibition assays  Can express, cannot purify |
|  |  |  |  |  |  |

| **Number** | **Primer Name** | **Primer Sequence** | **Ann.Temp. (C)** | **Size BP** | **Note** |
| --- | --- | --- | --- | --- | --- |
| LecB-F | LecB-ATG-Kpn1 | 5’ AGATCTGGGTACC ATGGCAACAC AAGGAGTGTT CA 3’ | 55 | 369 | For 12.7 kDa.  LecB protein purification.  Interaction Inhibition assays by ELISA.  Can express, can purify. |
| LecB-R | LecB-HIS+ Xho1 | 5’ TGGTGCTCGAG GCCGAGCGGCCAGTTGATCACCACG 3’ |  |  |  |
|  |  |  |  |  |  |
