# Supplementary material for: Marine prebiotics mediate decolonization of Pseudomonas aeruginosa from gut by inhibiting secreted virulence factor interactions with mucins and enriching Bacteroides population
Source: J Biomed Sci. 2023 Feb 2;30:9. doi: 10.1186/s12929-023-00902-w (PMC9896862; doi:10.1186/s12929-023-00902-w)
Supplement: Supplementary file 5 — Additional file 5: Table S4. Polysaccharides used in this study. [file 12929_2023_902_MOESM5_ESM.docx]

**Additional file 5: Table S4.**

Polysaccharides used in this study.

| **Biomolecules** | **Product code** | **Company** |
| --- | --- | --- |
|  |  |  |
| **Dextrans** |  |  |
| Dextran sulfate-500 kDa | D6001-10G | Sigma, USA |
| Dextran sulfate 6-11 kDa | D4911-10G |  |
| Dextran 450 kDa | 31392-10G |  |
| Dextran 9-10 kDa | D9260-10G |  |
| **Fucoidans and Mucin** |  |  |
| *Macrosystis pyrifera* | F8065-500MG |  |
| *Undaria pinnatifada* | F8315-500MG |  |
| *Fucus vesiculosus* crude | F5631-1G |  |
| *Ascophylllum nodusum* -nutri. 0.5% | Marinova ANF | Marinova, Australia |
| *A. nodusum*+*L. digitata* (PS-II) | PS111 | Dextra, UK |
| *Fucus vesiculosus* 95% | F8190-500MG | Sigma, USA |
| k-carrageenan | 22048-25G-F | Sigma, USA |
| *Fucus serratus* | OF09360-100G | Carbosynth, UK |
| *Fucus vesiculosus* – nutri. 0.5% | Marinova FVF | Marinova, Australia |
| *Laminaria digitata* | OF09361 | Carbosynth, UK |
| *L. japonica* | OF01606 | Carbosynth, UK |
| Porcine Gastric Mucin Type III | M1778 | Sigma, USA |
| Porcine Gastric Mucin Type II | M2378 | Sigma, USA |
| *A. nodusum* | OF09363 | Carbosynth, UK |
| **Glycosaminoglycans** |  |  |
| Chondroitin sulfate -Sigma | 27042-10G-F | Sigma, USA |
| **Alginates** |  |  |
| *A. nodusum* alginate | ALG102 | Elicityl, France |
| Alginic acid | A1112-100G | Sigma, USA |
| *L. japonica* alginate | ALG100 | Elicityl, France |
| *Azotobacter* spp. alginate | YB58638 | Carbosynth Limited,UK |
