# Supplementary material for: Marine prebiotics mediate decolonization of Pseudomonas aeruginosa from gut by inhibiting secreted virulence factor interactions with mucins and enriching Bacteroides population
Source: J Biomed Sci. 2023 Feb 2;30:9. doi: 10.1186/s12929-023-00902-w (PMC9896862; doi:10.1186/s12929-023-00902-w)
Supplement: Supplementary file 6 — Additional file 6: Table S5. Antibodies used in this study. [file 12929_2023_902_MOESM6_ESM.docx]

**Additional file 6: Table S5**

Antibodies used in this study.

| **Specific antibody** | **Antibody type** | **Product code** | **Company** |
| --- | --- | --- | --- |
| Blood Group A antigen Antibody (Z2A) FITC | mouse IgM | sc-69951 FITC | Santa Cruz Biotechnology, USA |
| Blood Group B Antigen Antibody (Z5H-2) FITC | mouse IgM | sc-69952 FITC | Santa Cruz Biotechnology, USA |
| Blood Group H ab antigen Antibody (87-N) FITC | mouse IgM | sc-52369 FITC | Santa Cruz Biotechnology, USA |
| Blood Group Lewis b Antibody (T218) | mouse IgM | sc-59470 | Santa Cruz Biotechnology, USA |
| Blood Group Lewis x Antibody (P12) | mouse IgM | sc-59471 | Santa Cruz Biotechnology, USA |
| Blood Group Lewis y Antibody (A70-C/C8) | mouse IgM | sc-59472 | Santa Cruz Biotechnology, USA |
| PSGL-1 Antibody (HECA-452)  (sialyl Lewis-X, 6’sulfo sialyl Lewis-X, sialyl Lewis-A) | rat IgM | sc-53514 | Santa Cruz Biotechnology, USA |
| Sialyl Lewis A Antibody (121SLE) | mouse IgM | MA1-19211 | Thermofisher, USA |
| Anti-Blood Group Lewis y Antibody [F3] | mouse IgM | ab3359 | Abcam, USA |
| Anti-Blood Group B Antigen Antibody | mouse IgM | ab2524 | Abcam, USA |
| Thomsen-Friedenreich Antibody (A78-G/A7) | mouse IgM | sc-59394 | Santa Cruz Biotechnology, USA |
| Goat Anti-Rat IgM mu chain (HRP) | IgG | ab97180 | Abcam, USA |
| SouthernBiotech Mouse Anti-Rat IgM (mu chain specific) HRP | IgG1 k | [OB3080-05](https://www.fishersci.com/shop/products/southernbiotech-mouse-anti-rat-igm-mu-chain-specific-5/ob308005) | [Fisher Scientific](https://www.fishersci.com/us/en/scientific-products/special-offers-and-programs/fisher-scientific-safety-services.html) |
| Peroxidase AffiniPure Rabbit Anti-Mouse IgM, µ Chain Specific | Whole IgG | 315-035-020 | Jackson ImmunoResearch Inc. USA |
| Peroxidase AffiniPure Goat Anti-Human IgM, Fc5μ fragment specific | Whole IgG | 09-035-129 | Jackson ImmunoResearch Inc. USA |
| Anti-6x Histidine tag antibody | Rabbit  IgG | HIT001R | Bioman, Taiwan |
