# Supplementary material for: Marine prebiotics mediate decolonization of Pseudomonas aeruginosa from gut by inhibiting secreted virulence factor interactions with mucins and enriching Bacteroides population
Source: J Biomed Sci. 2023 Feb 2;30:9. doi: 10.1186/s12929-023-00902-w (PMC9896862; doi:10.1186/s12929-023-00902-w)
Supplement: Supplementary file 7 — Additional file 7: Table S6. Lectins used in this study. [file 12929_2023_902_MOESM7_ESM.docx]

**Additional file 7: Table S6**

Lectins used in this study.

| **Lectin Symbol** | **Lectin name** | **Source** | **Ligand motif** | **Product code** | **Company** |
| --- | --- | --- | --- | --- | --- |
| **Mannose binding lectins** | | | |  | Vector Labs, USA |
| **ConA** | Concanavalin A | *Canavalia ensiformis* | α-D-mannosyl and α-D-glucosyl residues branched α-mannosidic structures (high α-mannose type, or hybrid type and biantennary complex type N-Glycans) | B1005 |  |
| **GNA** | Snowdrop lectin | *Galanthus nivalis* | α 1-3 and α 1-6 linked high mannose structures | B1245 |  |
| **Galactose N-acetylgalactosamine binding lectins** | | | |  |  |
| **DBA** | *Dolichos biflorus* agglutinin | *Dolichos biflorus* | α -GalNAc | B-1035 |  |
| **PNA** | Peanut agglutinin | *Arachis hypogaea* | Galβ1-3GalNAcα1-Ser/Thr (T-Antigen) | FL-1071 |  |
| **N-acetylglucosamine binding lectins** | | | |  |  |
| **WGA** | Wheat Germ Agglutinin, WGA | *Triticum vulgaris* | GlcNAcβ1-4GlcNAcβ1-4GlcNAc, Neu5Ac (sialic acid) | B-1025 |  |
| **DSL** | *Datura stramonium* lectin | *Datura stramonium* | GlcNAc(β1,4)GlcNAc oligomers, Gal(β1,4)GlcNAc | B-1185 |  |
| **N-acetylneuraminic acid binding lectins** | | | |  |  |
| **SNA** | Elderberry lectin | *Sambucus nigra* | Neu5Acα2-6Gal(NAc)-R | B-1305 |  |
| **MAL** | *Maackia amurensis* leukoagglutinin | *Maackia amurensis* | Neu5Ac/Gcα2,3Galβ1,4Glc(NAc) | B-1265 |  |
| **Fucose binding lectins** | | | |  |  |
| **UEA** | *Ulex europaeus* agglutinin | *Ulex europaeus* | Fucα1-2Gal-R | B-1065 |  |
| **AAL** | *Aleuria aurantia* lectin | *Aleuria aurantia* | Fucα1-2Galβ1-4(Fucα1-3/4)Galβ1-4GlcNAc, R2-GlcNAcβ1-4(Fucα1-6)GlcNAc-R1 | B-1395 |  |
| **LTL** | *Lotus tetragonolobus* lectin | *Lotus tetragonolobus* | α-1,2 Fuc; Fuc (α-1,3) GlcNAc | B-1325 |  |
|  |  |  |  |  |  |
