# Supplementary material for: Marine prebiotics mediate decolonization of Pseudomonas aeruginosa from gut by inhibiting secreted virulence factor interactions with mucins and enriching Bacteroides population
Source: J Biomed Sci. 2023 Feb 2;30:9. doi: 10.1186/s12929-023-00902-w (PMC9896862; doi:10.1186/s12929-023-00902-w)
Supplement: Supplementary file 8 — Additional file 8: Fig. S1. Haemagglutinin domains (HAD) present at N-terminus of TpsA, Type V secretion system proteins in Pseudomonas aeruginosa. Six different TpsA proteins can be found in P. aeruginosa PA14 strain. Haemagglutinin domains (HAD) are very well conserved in all the T5SS proteins. Both Tps1 and Tps2 protein sequences can be found in genomes of > 80 strains in Pseudomonas Genome Database (PGDB). Images were generated by BLAST program http://blast.ncbi.nlm.nih.gov/Blast.cgi. [file 12929_2023_902_MOESM8_ESM.docx]

**Additional file 8: Figure S1.**

Haemagglutinin domains (HAD) present at N-terminus of TpsA, Type V secretion system proteins in *Pseudomonas aeruginosa*. Six different TpsA proteins can be found in P. aeruginosa PA14 strain. Haemagglutinin domains (HAD) are very well conserved in all the T5SS proteins. Both Tps1 and Tps2 protein sequences can be found in genomes of >80 strains in Pseudomonas Genome Database (PGDB). Images were generated by BLAST program <http://blast.ncbi.nlm.nih.gov/Blast.cgi>.
