# Supplementary material for: Marine prebiotics mediate decolonization of Pseudomonas aeruginosa from gut by inhibiting secreted virulence factor interactions with mucins and enriching Bacteroides population
Source: J Biomed Sci. 2023 Feb 2;30:9. doi: 10.1186/s12929-023-00902-w (PMC9896862; doi:10.1186/s12929-023-00902-w)
Supplement: Supplementary file 9 — Additional file 9: Figure S2. Preliminary screening for interaction of TpsA-NT-HAD with different substrates coated on the microliter plate. Porcine gastric mucin type III (Sigma) showed high reactivity in the ELISA assay. Alfa glycoprotein (AGP), human ovarian cyst mucin (HOC), bovine submaxillary mucin (BSM), pig submaxillary mucin (PSM). Numbers indicate fractions. Porcine gastric mucin #4, a blood group A + H substance, that was derived from crude Porcine stomach mucin. Treatment of mucin #4 with HCl (pH 2.0, 90 min, 100 °C) yields Porcine gastric mucin #9, while acid hydrolysis (pH 1.5, 100 °C, 2 and 5 h) gives Porcine gastric mucins #14 and #21, respectively [12]. [file 12929_2023_902_MOESM9_ESM.docx]

**Additional file 9: Figure S2.**

Preliminary screening for interaction of TpsA-NT-HAD with different substrates coated on the microliter plate. Porcine gastric mucin type III (Sigma) showed high reactivity in the ELISA assay. Alfa glycoprotein (AGP), human ovarian cyst mucin (HOC), bovine submaxillary mucin (BSM), pig submaxillary mucin (PSM). Numbers indicate fractions. Porcine gastric mucin #4, a blood group A + H substance, that was derived from crude Porcine stomach mucin. Treatment of mucin #4 with HCl (pH 2.0, 90 min, 100°C) yields Porcine gastric mucin #9, while acid hydrolysis (pH 1.5, 100°C, 2 and 5 hrs) gives Porcine gastric mucins #14 and #21, respectively [12].
