# Supplementary material for: Marine prebiotics mediate decolonization of Pseudomonas aeruginosa from gut by inhibiting secreted virulence factor interactions with mucins and enriching Bacteroides population
Source: J Biomed Sci. 2023 Feb 2;30:9. doi: 10.1186/s12929-023-00902-w (PMC9896862; doi:10.1186/s12929-023-00902-w)
Supplement: Supplementary file 10 — Additional file 10: Table S7. IC50 values of polysaccharides to inhibit LecB-PA01 monomer from binding to Porcine Gastric Mucin Type III obtained by ELISA. [file 12929_2023_902_MOESM10_ESM.docx]

**Additional file 10: Table S7**

IC50 values of polysaccharides to inhibit LecB-PA01 monomer from binding to Porcine Gastric Mucin Type III obtained by ELISA

| **Inhibitors** | **IC50**  **average µg/mL** |
| --- | --- |
|  |  |
| **Dextrans** |  |
| Dextran sulfate-500 kDa | NSI |
| Dextran sulfate 6-11 kDa | NSI |
| Dextran 450 kDa | NSI |
| Dextran 9-10 kDa | NSI |
| **Fucoidans and Mucin** |  |
| *Macrosystis pyrifera* | NSI |
| *Undaria pinnatifada* | NSI |
| *Fucus vesiculosus* crude | NSI |
| *Ascophyllum nodusum* nutri. 0.5% | NSI |
| *A. nodusum*+*L. digitata* (PS-II) | NSI |
| *Fucus vesiculosus* 95% | NSI |
| k-carrageenan | NSI |
| *Fucus serratus* | NSI |
| *F. vesiculosus* Aus. 30kDa | NSI |
| *Fucus vesiculosus* – nutri. 0.5% | NSI |
| *Laminaria digitata* | NSI |
| *L. japonica* | NSI |
| Porcine Gastric Mucin Type III | 255 |
| *A. nodusum* | NSI |
| **Glycosaminoglycans** |  |
| Chondroitin sulfate -Sigma | NSI |
| **Alginates** |  |
| *A. nodusum* alginate | NSI |
| Alginic acid | NSI |
| *L. aponica* alginate | NSI |
| *Azotobacter* spp. alginate | NSI |
|  |  |

Note: NSI; no significant inhibition (IC50> 1000µg/mL), Fuc; fucose, nutri.; nutritional grade fucoidan*.* The IC50 values were estimated by using a free online program from AAT Bioquest (https://www.aatbio.com/tools/ic50-calculator).
