# Supplementary material for: Marine prebiotics mediate decolonization of Pseudomonas aeruginosa from gut by inhibiting secreted virulence factor interactions with mucins and enriching Bacteroides population
Source: J Biomed Sci. 2023 Feb 2;30:9. doi: 10.1186/s12929-023-00902-w (PMC9896862; doi:10.1186/s12929-023-00902-w)
Supplement: Supplementary file 11 — Additional file 11: Figure S3. Binding of blood group antigen-specific antibodies to porcine gastric mucin type III (PGM), Fucus vesiculosus fucoidan (FV) and Fucus serratus fucoidan (FS). TpsA-NT-HAD indicates N-terminal hemagglutinin domain in TpsA1 and TpsA2 proteins. Representative results for interaction between antibodies and immobilized PGM, FV and FS. A color gradient heat map, with high reactivity (Red) to no reactivity (Blue) based on lectin ELISA OD values, has been applied to the well values. Antibodies used in this study are shown in Additional file 6: Table S5. [file 12929_2023_902_MOESM11_ESM.docx]

**
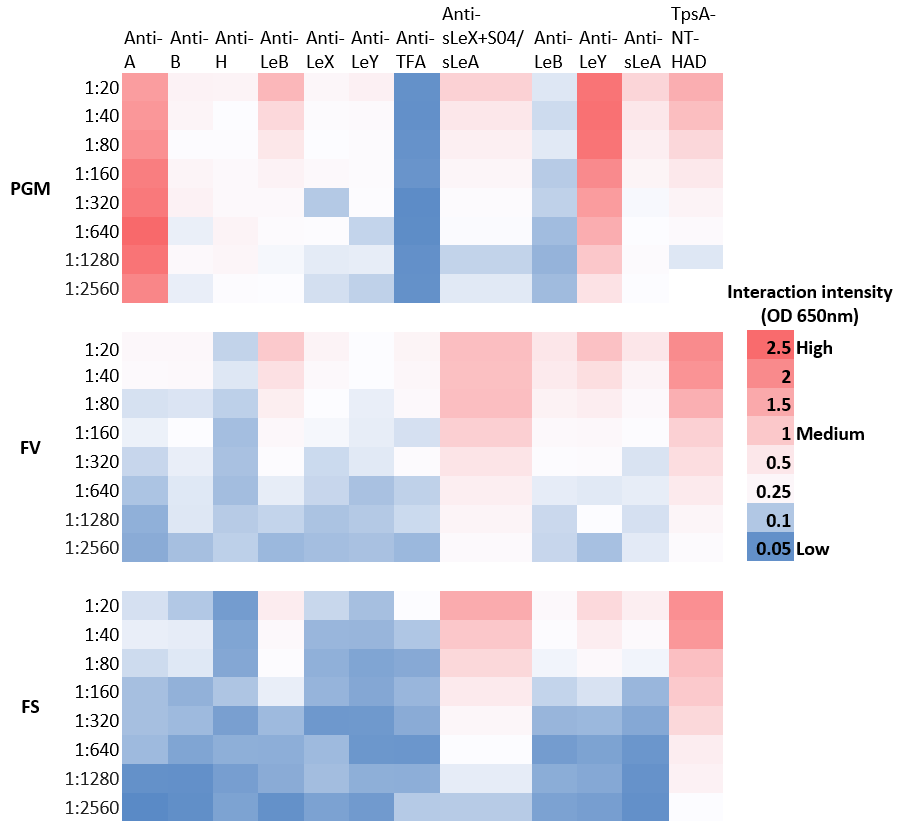
**

**Additional file 11: Figure S3.**

Binding of blood group antigen-specific antibodies to porcine gastric mucin type III (PGM), *Fucus vesiculosus* fucoidan (FV) and *Fucus serratus* fucoidan (FS)*.* TpsA-NT-HAD indicates N-terminal hemagglutinin domain in TpsA1 and TpsA2 proteins. **Representative results for interaction between antibodies and immobilized PGM, FV and FS. A color gradient heat map, with high reactivity (Red) to no reactivity (Blue) based on lectin ELISA OD values, has been applied to the well values. Antibodies used in this study are shown in Additional file 6:** Table S5**.**
