# Supplementary material for: Marine prebiotics mediate decolonization of Pseudomonas aeruginosa from gut by inhibiting secreted virulence factor interactions with mucins and enriching Bacteroides population
Source: J Biomed Sci. 2023 Feb 2;30:9. doi: 10.1186/s12929-023-00902-w (PMC9896862; doi:10.1186/s12929-023-00902-w)
Supplement: Supplementary file 12 — Additional file 12: Figure S4. Binding of specific plant lectins fucoidans, Fucus vesiculosus crude (FVC), Fucus vesiculosus 95% (FVP), Fucus serratus fucoidan (FS) and Ascophyllum nodusum (AN). Previously well-characterized porcine gastric mucin type III (PGM) was used as s positive control. Representative results for interaction between plant lectins and immobilized PGM, FVC, FVP, FS and AN. A color gradient heat map, with high reactivity (Red) to no reactivity (Blue) based on lectin ELISA OD values, has been applied to the well values. Note: Dilutions of lectins AAL and ConA in buffer were fourfold. Reactivity was low with other lectins including, SNA, PNA, GNA, DFL, WGA, SBA, DBA. Carbohydrate specificities of major plant lectins used in this study are shown in Additional file 7: Table S6. [file 12929_2023_902_MOESM12_ESM.docx]

**
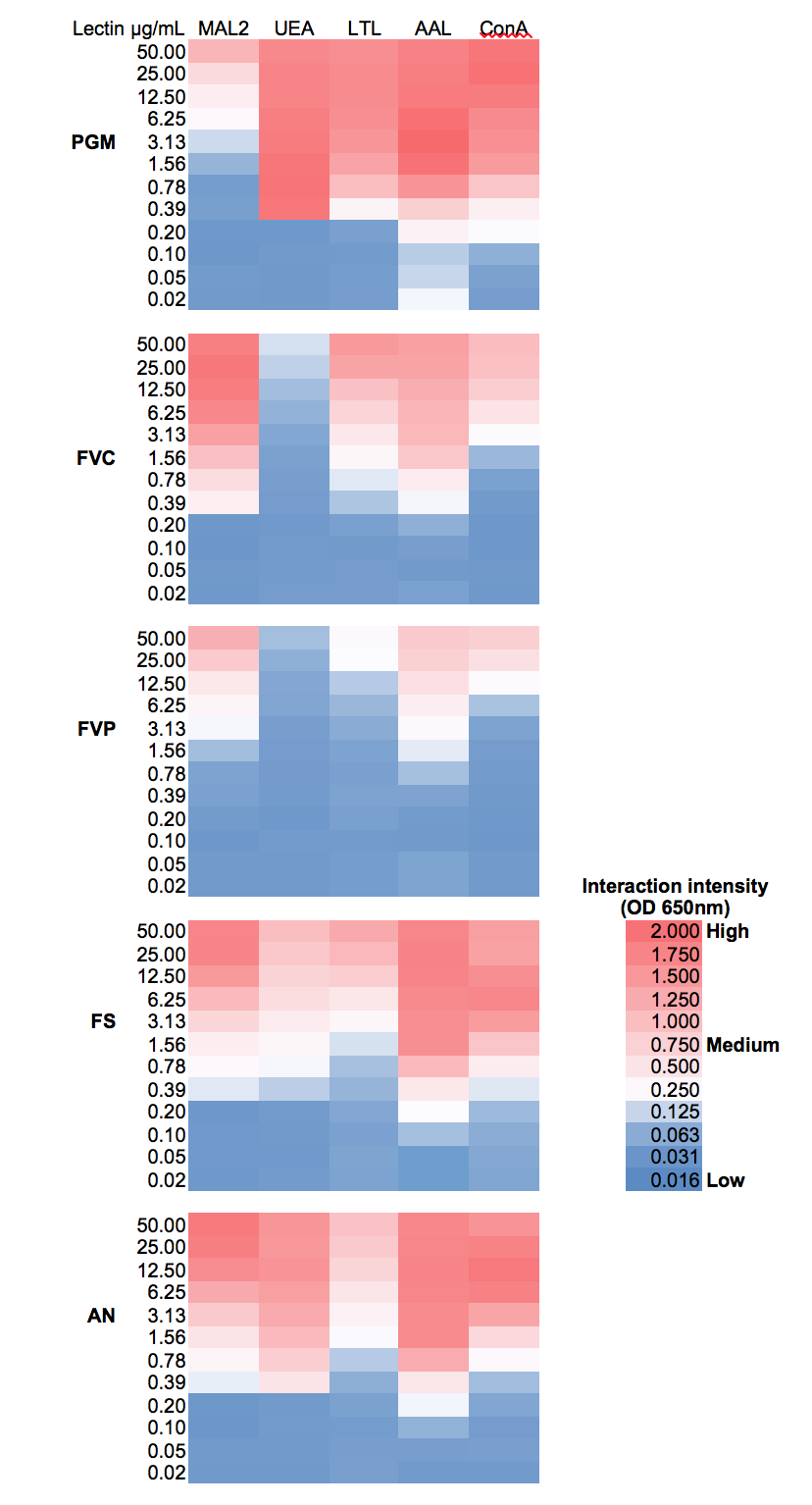
**

**Additional file 12: Figure S4.**

Binding of specific plant lectins fucoidans, *Fucus vesiculosus* crude (FVC), *Fucus vesiculosus* 95% (FVP), *Fucus serratus* fucoidan (FS) and *Ascophyllum nodusum* (AN). Previously well-characterized porcine gastric mucin type III (PGM) was used as s positive control. **Representative results for interaction between plant lectins and immobilized PGM,** FVC, FVP, FS and AN. **A color gradient heat map, with high reactivity (Red) to no reactivity (Blue) based on lectin ELISA OD values, has been applied to the well values. Note: Dilutions of lectins AAL and ConA in buffer were 4-fold. Reactivity was low with other lectins including, SNA, PNA, GNA, DFL, WGA, SBA, DBA. Carbohydrate specificities of major plant lectins used in this study are shown in Additional file 7:** Table S6**.**
