# Supplementary material for: Marine prebiotics mediate decolonization of Pseudomonas aeruginosa from gut by inhibiting secreted virulence factor interactions with mucins and enriching Bacteroides population
Source: J Biomed Sci. 2023 Feb 2;30:9. doi: 10.1186/s12929-023-00902-w (PMC9896862; doi:10.1186/s12929-023-00902-w)
Supplement: Supplementary file 13 — Additional file 13: Figure S5. Inhibition of P. aeruginosa S8 adhesion to Caco-2 intestinal cells by Fucus vesiculosus fucoidan (FV) (A) and Fucus serratus fucoidan (FS) (B). P. aeruginosa S8 were pre-incubated with fucoidans in 250-μl DMEM medium for 1 h at 37 °C in 5% CO2 and added to monolayers of Caco-2 cells with an MOI of 10 (2 X 105 cells) and incubated for additional 1 h at 37 °C in 5% CO2. Significant differences using Chi Square test in adhesion in comparison to the adhesion of bacteria incubated without fucoidan are indicated by asterisk (**, P ≤ 0.01; ***, P ≤ 0.001). MOI: Multiplicity of infection (Bacterium: host cell). [file 12929_2023_902_MOESM13_ESM.docx]

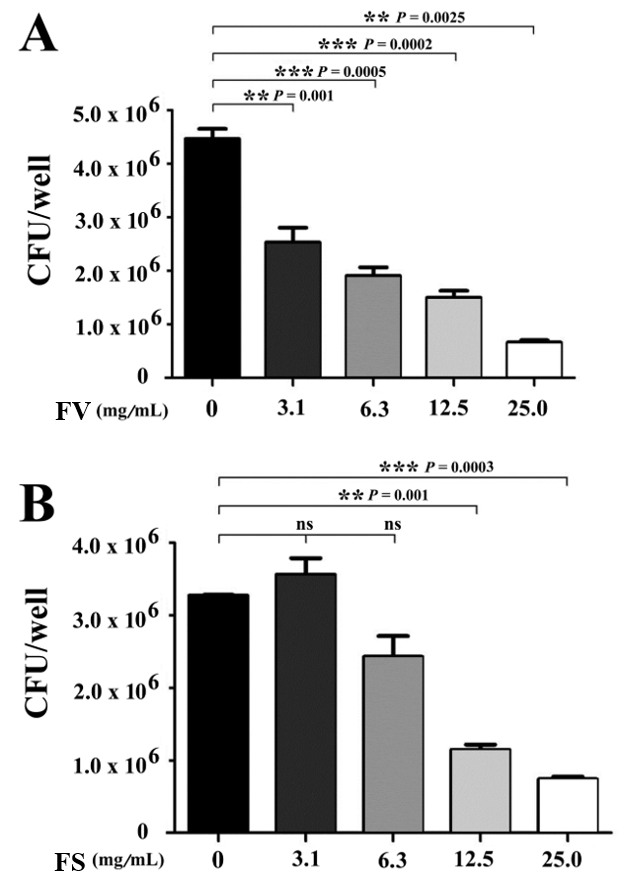


**Additional file 13: Figure S5.**

**Inhibition of** *P. aeruginosa* S8 adhesion to Caco-2 intestinal cells **by** *Fucus vesiculosus* fucoidan (FV) (A) and *Fucus serratus* fucoidan (FS) (B). *P. aeruginosa* S8 were pre-incubated with fucoidans in 250-μl DMEM medium for 1 h at 37°C in 5% CO_2_ and added to monolayers of Caco-2 cells with an MOI of 10 (2 X 10^5^ cells) and incubated for additional 1 hour at 37°C in 5% CO_2._ Significant differences using Chi Square test in adhesion in comparison to the adhesion of bacteria incubated without fucoidan are indicated by asterisk (**, *P ≤* 0.01; ***, *P ≤* 0.001). MOI: Multiplicity of infection (Bacterium: host cell).
