# Supplementary material for: Marine prebiotics mediate decolonization of Pseudomonas aeruginosa from gut by inhibiting secreted virulence factor interactions with mucins and enriching Bacteroides population
Source: J Biomed Sci. 2023 Feb 2;30:9. doi: 10.1186/s12929-023-00902-w (PMC9896862; doi:10.1186/s12929-023-00902-w)
Supplement: Supplementary file 14 — Additional file 14: Figure S6. Inhibition of bacterial adhesion to Caco-2 intestinal cells in the comparison of fucoidans with monosaccharides. P. aeruginosa S8 (A) and ESBL-producing ST131 E. coli as well as Non-ESBL-producing ST131 E. coli (B) were tested. Each test was pre-incubated with for 1 h at 37 °C in 5% CO2 and added to monolayers of Caco-2 cells (2 X 105 cells) and incubated for additional 1 h at 37 °C in 5% CO2. Fucoidans (FV, Fucus vesiculosus; FS, Fucus serratus; LJ, Laminaria Japonicia) were used (each 12.5 mg/mL). Monosaccharides (10 mM), including dextran (Dex), mannose (Man), glucose (Glc), D-fucose (D-F), were tested. Significant difference in inhibition of bacterial adhesion with fucoidan treatment compared to the adhesion without fucoidan treatment is indicated by asterisk (**, P ≤ 0.01; ***, P ≤ 0.001). ESBL: extended-spectrum β-lactamase; ns: no significance. [file 12929_2023_902_MOESM14_ESM.docx]

**
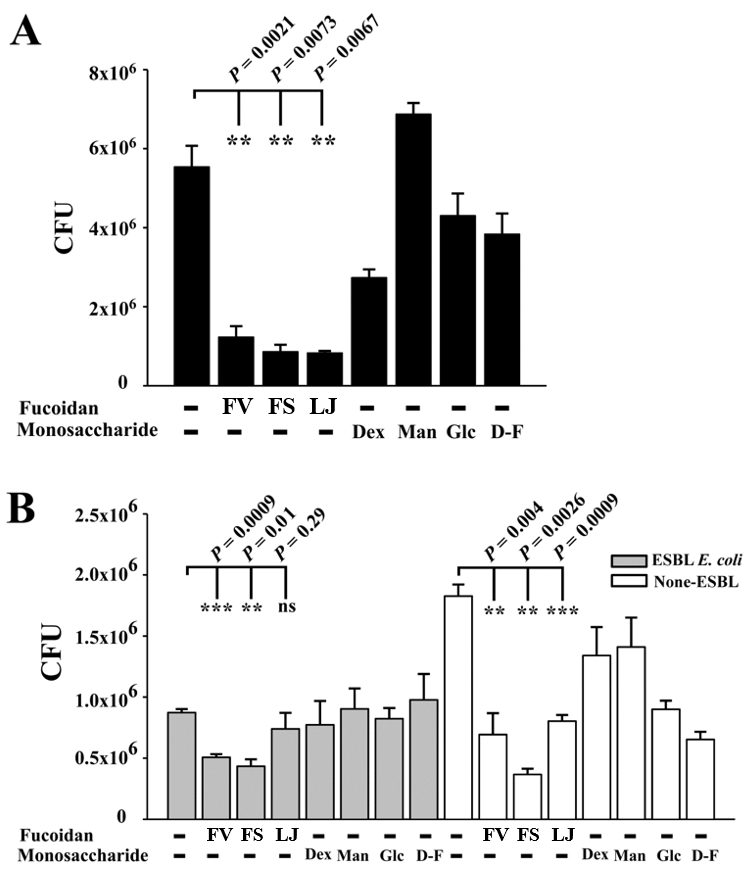
**

**Additional file 14: Figure S6.**

**Inhibition** of bacterial adhesion to Caco-2 intestinal cells **in the comparison of fucoidans with monosaccharides**. *P. aeruginosa* S8 (**A**) and ESBL-producing ST131 *E. coli* as well as Non-ESBL-producing ST131 *E. coli* (**B**) were tested. Each test was pre-incubated with for 1 h at 37 °C in 5% CO_2_ and added to monolayers of Caco-2 cells (2 X 10^5^ cells) and incubated for additional 1 hour at 37 °C in 5% CO_2_. Fucoidans (FV, *Fucus vesiculosus*; FS, *Fucus serratus*; LJ, *Laminaria Japonicia*) were used (each 12.5 mg/mL)*.* Monosaccharides (10 mM), including dextran (Dex), mannose (Man), glucose (Glc), D-fucose (D-F), were tested. Significant difference in **inhibition** of bacterial adhesion with fucoidan treatment compared to the adhesion without fucoidan treatment is indicated by asterisk (**, *P ≤* 0.01; ***, *P ≤* 0.001). ESBL: extended-spectrum β-lactamase; ns: no significance.
