# Supplementary material for: Marine prebiotics mediate decolonization of Pseudomonas aeruginosa from gut by inhibiting secreted virulence factor interactions with mucins and enriching Bacteroides population
Source: J Biomed Sci. 2023 Feb 2;30:9. doi: 10.1186/s12929-023-00902-w (PMC9896862; doi:10.1186/s12929-023-00902-w)
Supplement: Supplementary file 15 — Additional file 15: Figure S7. Sulfate content of fucoidan samples after thermal treatment (T) at 80 °C. A, 10 and B, 60 min; and after C, overnight acid hydrolysis at 80 °C to liberate sulfate ions in thermally treated samples. HMW-untreated high molecular weight fucoidan Fv (Fucus vesiculosus 95%) as control. T-purified – thermally treated fucoidan Fv, its filtrate and mixture of both (mix). T10 and T60- purified Fv (Fucus vesiculosus 95%) obtained after 10 or 60 min at 80 °C, purified and hydrolyzed in HCl overnight at 80 °C to release sulfate ions. [file 12929_2023_902_MOESM15_ESM.docx]

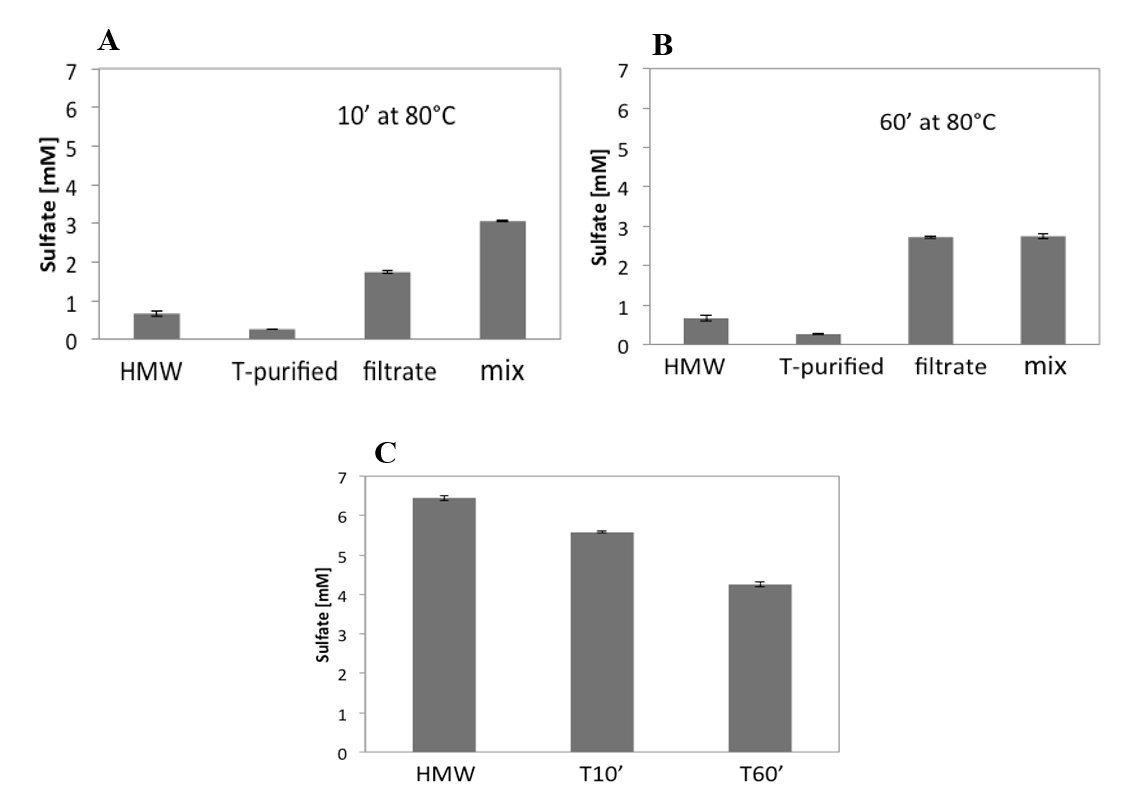


**Additional file 15: Figure S7.**

Sulfate content of fucoidan samples after thermal treatment (T) at 80°C. **A**, 10 and **B**, 60 min; and after **C**, overnight acid hydrolysis at 80°C to liberate sulfate ions in thermally treated samples. HMW-untreated high molecular weight fucoidan Fv (*Fucus vesiculosus* 95%) as control. T-purified – thermally treated fucoidan Fv, its filtrate and mixture of both (mix). T10 and T60- purified Fv (*Fucus vesiculosus* 95%) obtained after 10 or 60 min at 80°C, purified and hydrolyzed in HCl overnight at 80°C to release sulfate ions.
