# Supplementary material for: Marine prebiotics mediate decolonization of Pseudomonas aeruginosa from gut by inhibiting secreted virulence factor interactions with mucins and enriching Bacteroides population
Source: J Biomed Sci. 2023 Feb 2;30:9. doi: 10.1186/s12929-023-00902-w (PMC9896862; doi:10.1186/s12929-023-00902-w)
Supplement: Supplementary file 16 — Additional file 16: Figure S8. Fucose content of fucoidan Fv (Fucus vesiculosus 95%) samples after thermal (T) and thermal treatment and acid (T + A) treatment at 80 °C. A,D 10 and B,E 60 min; and after C,F overnight acid hydrolysis at 80 °C to liberate sulfate ions and sugar monomers. HMW-untreated high molecular weight fucoidan Fv (Fucus vesiculosus 95%) as control. [file 12929_2023_902_MOESM16_ESM.docx]

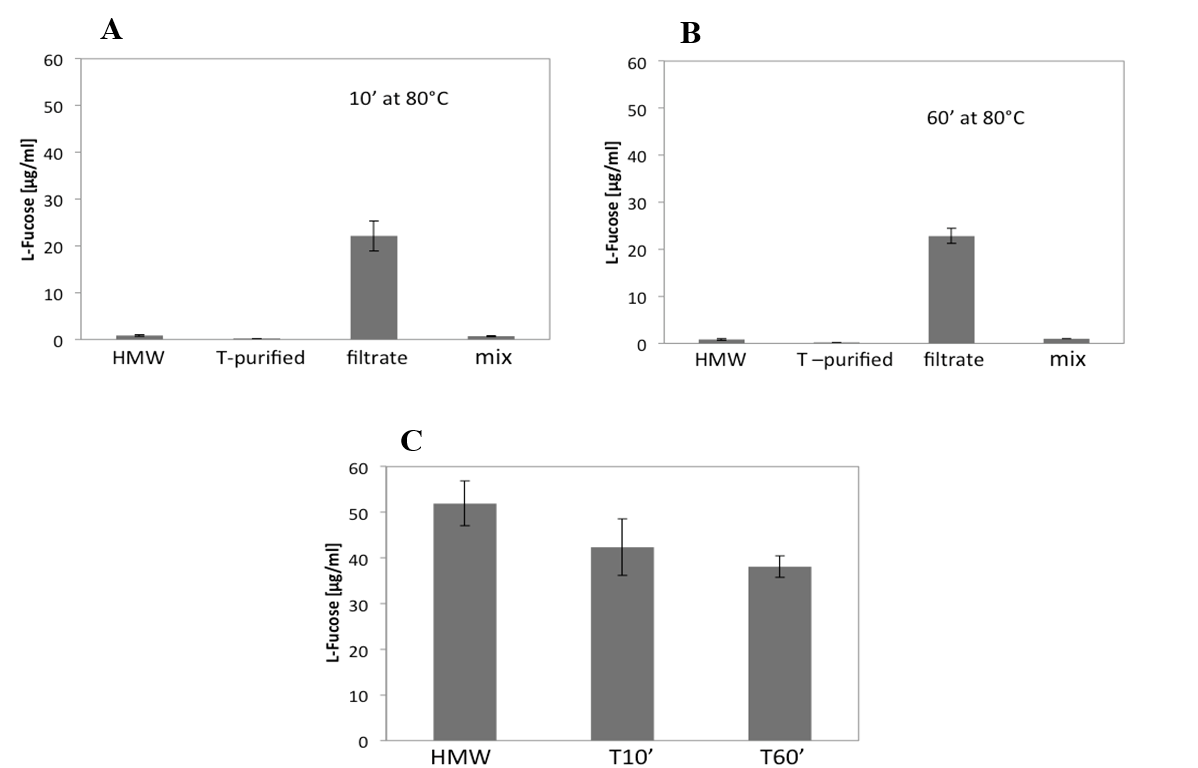


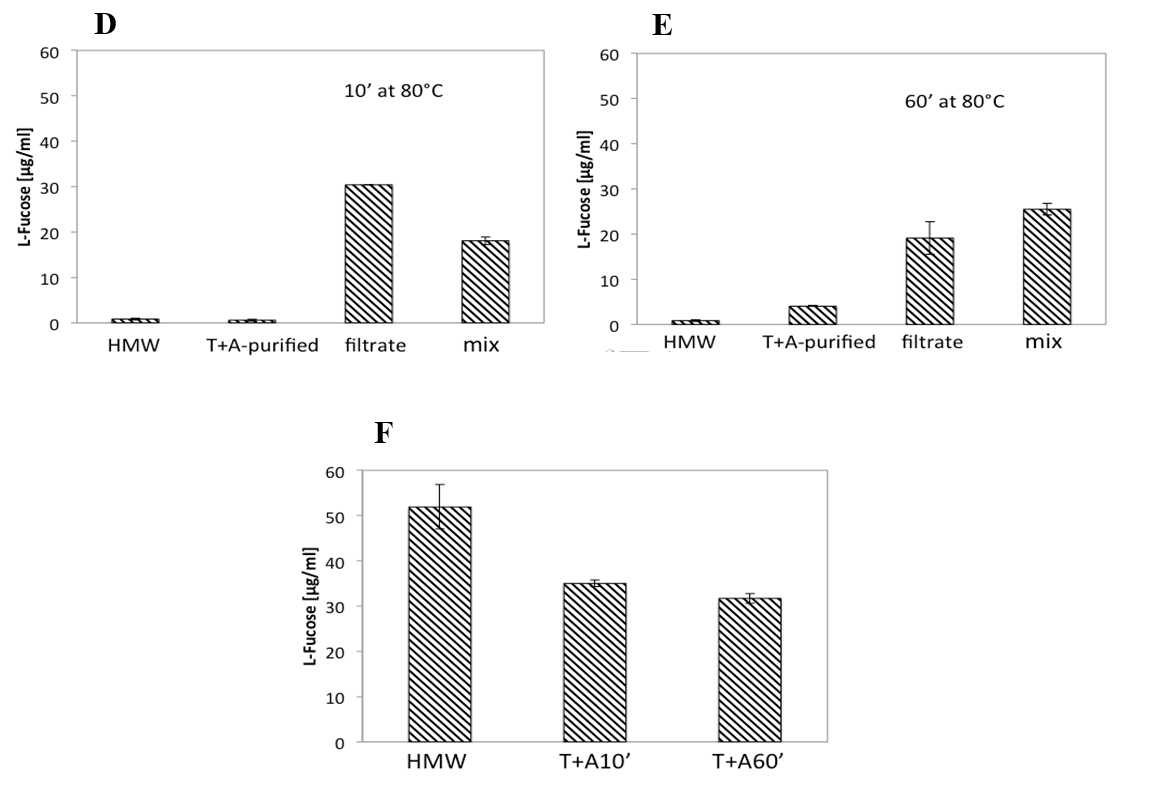


**Additional file 16: Figure S8.**

Fucose content of fucoidan Fv (*Fucus vesiculosus* 95%) samples after thermal (T) and thermal treatment and acid (T+A) treatment at 80°C. **A**,**D** 10 and **B**,**E** 60 min; and after **C**,**F** overnight acid hydrolysis at 80°C to liberate sulfate ions and sugar monomers. HMW-untreated high molecular weight fucoidan Fv (*Fucus vesiculosus* 95%) as control.
