# Supplementary material for: Marine prebiotics mediate decolonization of Pseudomonas aeruginosa from gut by inhibiting secreted virulence factor interactions with mucins and enriching Bacteroides population
Source: J Biomed Sci. 2023 Feb 2;30:9. doi: 10.1186/s12929-023-00902-w (PMC9896862; doi:10.1186/s12929-023-00902-w)
Supplement: Supplementary file 17 — Additional file 17: Table S8. Significant reductions in fecal P. aeruginosa load among the study groups during the study period. [file 12929_2023_902_MOESM17_ESM.docx]

**Additional file 17: Table S8**

Significant reductions in fecal *P. aeruginosa* load among the study groups during the study period.

|  | **Water** | **Fucoidan**  ***F. vesiculosus*** | **Fucoidan**  ***A. nodusum*** |
| --- | --- | --- | --- |
|  |  | **P- value** |  |
| Days1 VS Days 3 | 0.012* | 0.038* | 0.169 |
| Days1 VS Days 5 | 0.208 | 0.051 | 0.022* |
| Days1 VS Days 7 | 0.674 | 0.021* | 0.028* |
| Days1 VS Days 14 | 0.123 | 0.008** | 0.028* |
| Days1 VS Days 16 | 0.123 | 0.008** | 0.022* |
| Days1 VS Days 19 | 0.012* | 0.008** | 0.017* |
| Days1 VS Days 22 | 0.012* | 0.008** | 0.005** |
| Days1 VS Days 30 | 0.012* | 0.008** | 0.005** |

Statistical significance was determined using Wilcoxon signed-rank test. NS, not significant, **P* < 0.05, ***P* < 0.01.
