# Supplementary material for: Marine prebiotics mediate decolonization of Pseudomonas aeruginosa from gut by inhibiting secreted virulence factor interactions with mucins and enriching Bacteroides population
Source: J Biomed Sci. 2023 Feb 2;30:9. doi: 10.1186/s12929-023-00902-w (PMC9896862; doi:10.1186/s12929-023-00902-w)
Supplement: Supplementary file 18 — Additional file 18: Figure S9. Decolonization and recolonization in individual mice after fucoidan-treatment. Time lines of fecal shedding tests were scheduled for 30 days, including 14 days treatment of fucoidans Fv (Fucus vesiculosus) and FA (Ascophyllum nodusum) after challenge of Shanghai-fever strain P. aeruginosa S8. Negative control (NC) was the mouse challenged with water alone. Oral challenge of P. aeruginosa (1.0 × 10^7^ CFU) was applied for three days from Day-2 to Day 0. Fucoidans FVF, and ANF (0.5% in water, w/v) were used to feed mice (10 mice per each group) from Day-4 to Day + 14. [file 12929_2023_902_MOESM18_ESM.docx]

**Additional file 18: Figure S9.**

**Decolonization and recolonization in individual mice after fucoidan-treatment.** Time lines of fecal shedding tests were scheduled for 30 days, including 14 days treatment of fucoidans FV (*Fucus vesiculosus*) and FA (*Ascophyllum nodusum*) after challenge of Shanghai-fever strain *P. aeruginosa* S8. Negative control (NC) was the mouse challenged with water alone. Oral challenge of *P. aeruginosa* (1.0 x 10^7^ CFU) was applied for three days from Day -2 to Day 0. **Fucoidans FVF, and ANF (0.5% in water, w/v) were used to feed** mice (10 mice per each group) from Day -4 to Day +14.
