# Supplementary material for: Marine prebiotics mediate decolonization of Pseudomonas aeruginosa from gut by inhibiting secreted virulence factor interactions with mucins and enriching Bacteroides population
Source: J Biomed Sci. 2023 Feb 2;30:9. doi: 10.1186/s12929-023-00902-w (PMC9896862; doi:10.1186/s12929-023-00902-w)
Supplement: Supplementary file 20 — Additional file 20: Figure S11. Changes in relative abundance at phylum level were compared between groups on post infection (P.I.) day 3, 7, 14 and 30. Differences between groups were calculated using a t-test, numbers above columns indicate P values (significant difference, P < 0.05). [file 12929_2023_902_MOESM20_ESM.docx]

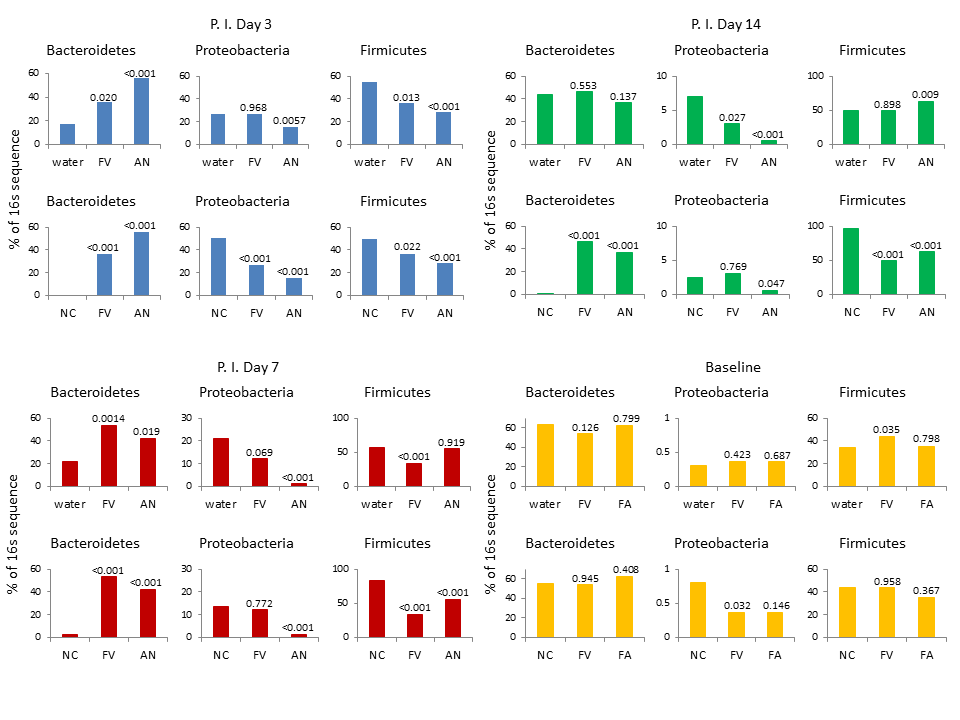
**Additional file 20: Figure S11.**

Changes in relative abundance at phylum level were compared between groups on post infection (P.I.) day 3, 7, 14 and 30. Differences between groups were calculated using a t-test, numbers above columns indicate *P* values (significant difference, *P* < 0.05).
